# What are the barriers and facilitators to seeking help for mental health in NHS doctors: a systematic review and qualitative study

**DOI:** 10.1186/s12888-022-04202-9

**Published:** 2022-09-07

**Authors:** Nadia Zaman, Khadeejah Mujahid, Fahmid Ahmed, Simran Mahmud, Hamza Naeem, Umar Riaz, Umayair Ullah, Benita Cox

**Affiliations:** Imperial College Business School, Exhibition Road, London, SW7 2AZ UK

**Keywords:** Mental health, Barriers, Facilitators, Help-seeking behaviour, NHS doctors, Systematic review, Qualitative study

## Abstract

**Background:**

The mental health of healthcare professionals is reaching a breaking point, and the COVID-19 pandemic has exacerbated current mental health issues to unprecedented levels. Whilst some research has been carried out on the barriers that doctors face when seeking mental health help, there is little research into factors which may facilitate seeking help. We aimed to expand the research base on factors which act as barriers to seeking help, as well as gain insight into facilitators of help-seeking behaviour for mental health in NHS doctors.

**Methods:**

We conducted a systematic literature review which identified the barriers and facilitators to seeking help for mental health in healthcare professionals. Following this, we conducted semi-structured interviews with 31 NHS doctors about their experiences with mental health services. Finally, through thematic analysis, key themes were synthesised from the data.

**Results:**

Our systematic literature review uncovered barriers and facilitators from pre-existing literature, of which the barriers were: preventing actions, self-stigma, perceived stigma, costs of seeking treatment, lack of awareness and availability of support, negative career implications, confidentiality concerns and a lack of time to seek help. Only two facilitators were found in the pre-existing literature, a positive work environment and availability of support services.

Our qualitative study uncovered additional barriers and facilitators, of which the identified barriers include: a negative workplace culture, lack of openness, expectations of doctors and generational differences. The facilitators include positive views about mental health, external confidential service, better patient outcomes, protected time, greater awareness and accessibility, open culture and supportive supervisors.

**Conclusion:**

Our study began by identifying barriers and facilitators to seeking mental health help in healthcare workers, through our systematic literature review. We contributed to these findings by identifying themes in qualitative data.. Our findings are crucial to identify factors preventing NHS doctors from seeking help for their mental health so that more can be done on a national, trust-wide and personal level to overcome these barriers. Likewise, further research into facilitators is key to encourage doctors to reach out and seek help for their mental health.

**Supplementary Information:**

The online version contains supplementary material available at 10.1186/s12888-022-04202-9.

## Background

The mental health of healthcare professionals (HCPs) is reaching a breaking point. A recent survey from the BMA into the mental health of doctors found that a quarter reported being diagnosed with a mental health condition at some point in their life, with 90% stating that their current work environment had contributed to their condition, to some extent [1]. Long, arduous and competitive training, as well as difficult working hours and a lack of time off work [2] can all contribute to burnout, a psychological syndrome characterised by ‘emotional exhaustion, feelings of cynicism and reduced personal accomplishment’ [3].

The COVID-19 pandemic has exacerbated these issues [4–6], with 43% of doctors surveyed stating that their work-related mental health issues had been worsened as a direct result [7].

In comparison to the general population, HCPs have some of the highest rates of work-related mental health issues [5, 8, 9] and the highest suicide rates of any occupational group in England and Wales [10]. This has been estimated to be two to five times that of the general population [11, 12].

HCPs also have different mental health needs, due to the unique nature and demands of their roles [13–15]. These include unrealistic expectations of being immune to mistakes or ill health, which can cause significant stress [16, 17].

One survey highlighted that 60% of doctors felt their mental health issues impacted their concentration, with research suggestingthat physician burnout is negatively correlated with patient quality of care and safety [18, 19]. This is cause for concern as studies have also shown doctors who are less engaged are significantly more prone to mistakes [20, 21]. It is estimated that more than £800 m was spent on mental health related absences during the Covid-19 pandemic equating to 3.7 million lost working days [22]. Arguably, funding would be better spent on helping doctors seek help the first instance [20].

HCPs often find themselves unsure of where to seek help for their own mental health [23]. As a result, thousands of staff members have left the NHS due to ill health [20, 22], with thousands more planning to leave due to pandemic-related stress, damaging an already resource constrained service [24].

Help-seeking behaviour (HSB) is defined by Rickwood and Thomas [25] as any action of actively “seeking assistance, in the form of guidance, treatment or support from healthcare services or from trusted people in the community”. Mental health HSB is specified as “an adaptive coping process which is the attempt to obtain external assistance to deal with a mental health concern” [25].

This includes two sources of help:

1. Formal—Any professional services designed to treat or support mental well-being.
2. Informal—Any action outside these appointed services that can be used to improve mental well-being e.g., speaking to friends and family or actions that help reduce mental stresses [26].

Although informal help is undoubtedly useful in helping with mental health concerns, they occur more spontaneously which makes them much more difficult to evaluate [27]. For the purposes of this study, we will focus on formal sources of help. As highlighted by Bach-Mortensen & Verboom [28] defining barriers and facilitators in research are important to ensure correct identification. The authors developed the definition for ‘barrier’ and ‘facilitator’ based on whether the supposed action complimented the definition of ‘mental health help-seeking behaviour’, as stated above by Rickwood and Thomas. For the purposes of this project, barriers and facilitators are defined as follows:

- Barrier—a circumstance or obstacle that keeps people from accessing mental health services.
- Facilitator—a circumstance or process that makes it easier for people to access mental health services.

### Aim of study

The overarching aim for this study is to understand the factors involved in HCP’s seeking help for their mental health. This would comprise 2 phases; a systematic literature review (SLR) and primary data collection.

The aim of the SLR) is to summarise and critically appraise the reported barriers and facilitators to mental health HSB in HCPs from existing literature. The factors are then categorised into broader themes. TFollowing this the qualitative study will then explore and develop the barriers faced by doctors when seeking help for their mental health and factors which facilitate this process.

Managing the mental ill-health of doctors is a challenge the NHS faces and will continue to face for years to come, hence understanding the barriers and facilitators to doctors seeking help could lead to the creation of services that are tailored to these individual factors, thereby encouraging help-seeking.

### Systematic literature review

Our preliminary research suggests that no SLR has previously been conducted to synthesise the literature on the barriers and facilitators to mental health HSB in HCPs. A broader search on HCPs rather than doctors only was conducted to increase the number of articles identified. Also, the stressors impacting doctors also seemed to impact other HCPs as well [17–19], suggesting their mental health HSB may also be similar.

SLR Question: What are the barriers and facilitators to the HSB of HCPs with regards to their mental health?

## Methods

### Search methodology

A scoping search was initially done using the PICO framework to identify the main keywords (Table 1), forming the final search terms.

**Table 1:** The PICO framework identifying the keywords

Population	HCPs
Intervention	Barriers or facilitator of HSB for mental health
Comparator	No barriers or facilitator of HSB for mental health
Outcome	Seeking help for mental health

Subsequently, a systematic search of the literature (see Additional File 1 for search string strategy) used the electronic databases MEDLINE (1946 – 2021), Exceptra Medica Database (EMBASE) (1947 – 2021), Health Management Information Consortium (HMIC) (1979 – 2021), APA PsycINFO (1806 – 2021) in February 2021. Additional articles were added by manually scanning reference lists of literature review papers, identified by the database searches.

Keywords were selected through an initial iterative search strategy in combination with identifying Medical Subject Headings (MeSH) from each database. The final search for MEDLINE, Embase, HMIC and APA PsycINFO included keywords relating to the following concepts: mental health, HSB and HCPs. Search results were limited to the English language.

### Eligibility criteria

Articles focussing on barriers or facilitators to mental health HSB were selected. The full inclusion and exclusion criteria for selecting the articles are outlined in Table 2.

**Table 2:** The inclusion and exclusion criteria for the SLR

Inclusion Criteria	Exclusion Criteria
English language	Not in the English language
Data collected from HCPs	Data not collected from HCPs
Articles related to HSB for mental health	Articles related to HSB for general health
Qualitative or quantitative studies, mixed methods studies	Literature reviews, theoretical studies, intervention studies, systematic reviews

### Study selection

Two thousand fifty-five articles were found across the four databases. After removing duplicates, the articles totalled 1480. These articles were filtered by initial screening of the title and abstract and then reviewing the full text of the articles using the exclusion criteria (Table 2). Any conflict on article inclusion was decided by group consensus. Thereafter, the full text of the articles was reviewed using the inclusion criteria (Table 2). Finally, the articles were critically appraised using the Centre for Evidence- Based Management (CEBMa) checklist and Critical Appraisal Skills Programme (CASP) (Additional file 4). CEBMa was used for cross-sectional and cohort studies and CASP was used for qualitative studies.

The selection process was undertaken by two researchers; pilot testing was conducted as part of the screening process in order to ensure consistency between researchers.

**Fig. 1:**
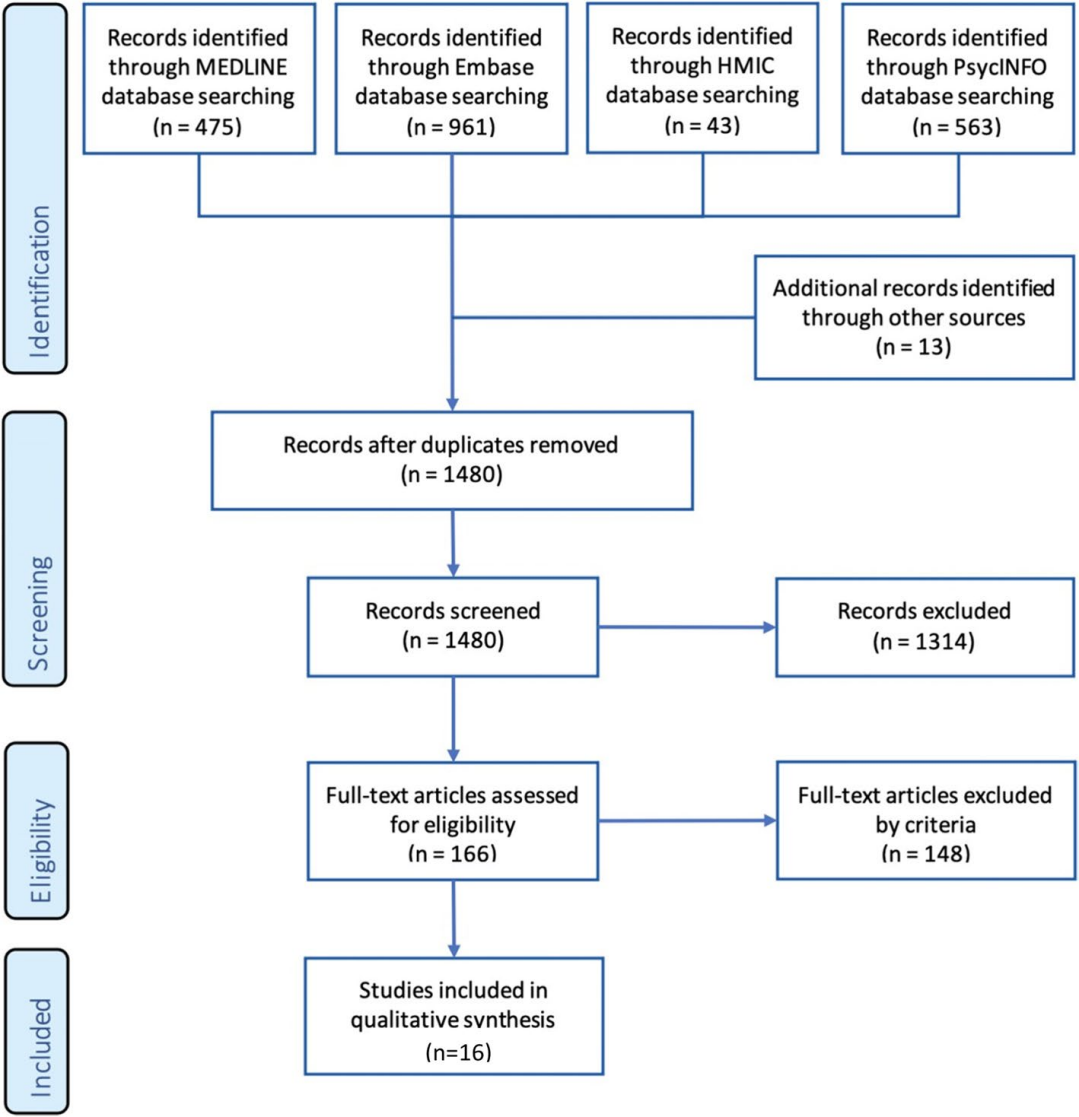
PRISMA flow diagram

The screening process has been outlined using the Preferred Reporting Items for Systematic Reviews and Meta-Analyses (PRISMA) flow diagram in Fig. 1.

### Data synthesis

The articles were tabulated, recording the author, year, study characteristics, study population, barriers and facilitators. The principal themes for the barriers and facilitators were identified from the quantitative data. Barriers and facilitators reported in qualitative studies were extracted via thematic synthesis.

## Results

### Summary of the studies

A total of 16 studies reporting barriers and/or facilitators were included in the review. The studies were conducted in the UK (*n* = 7), USA (*n* = 4), Australia (*n* = 2), South Africa (*n* = 1), Singapore (*n* = 1) and South Korea (*n* = 1). The methodology of the studies either adopted a quantitative approach using surveys (*n* = 12) or a qualitative approach using interviews (*n* = 4). Most studies focused on doctors (*n* = 14) and two studies focused on other HCPs. A summary of the articles, including sample size and characteristics, has been provided in Additional File 2. Summary of Findings.

**Fig. 2:**
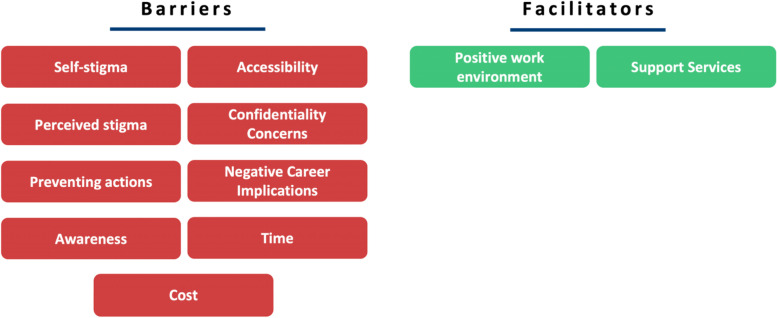
Themes identified in the SLR categorised into ‘Barriers’ and ‘Facilitators’

The themes found during the SLR are summarised in Fig. 2.

### Barriers to help-seeking behaviour for mental health

#### Confidentiality concerns

Confidentiality is a key concern that was cited in several studies [29–34]. Spiers et al. [32], found that doctors are concerned about negatively impacting the doctor-patient relationship, potentially due to doctors perceiving themselves as vulnerable [34, 35]. The study also found doctors are afraid colleagues could find out about their mental health condition, potentially impacting workplace relationships. Doctors may fear letting down colleagues who rely on them, by taking time off work [32, 34]. As the study was conducted on a small sample of GPs, this may not be representative of the wider HCP population [33, 36].

However, larger studies including White et al. [33] and Rees et al. [36] supported the finding that confidentiality was a barrier to seeking help across specialties. White et al. [33] found confidentiality to be the most prevalent cause for psychiatrists not seeking help (66.2%) and emphasised the need for specialist psychiatric services for doctors.

Adams et al. [34] found that confidentiality was an issue particularly amongst those with a previous diagnosis of depression. The findings are corroborated by Schwenk et al.’s [37] study of 5000 physicians that found those with moderate to severe depression were more likely to avoid seeking treatment due to confidentiality concerns.

The studies that cited confidentiality as a barrier to HSB were all in doctors, and do not necessarily represent other HCPs. Additionally, all the study results are self-reported, meaning the prevalence of confidentiality concerns may be under-reported due to anonymity concerns, [32, 36]. Despite this, confidentiality was still raised as a key barrier across these studies, emphasising it as a prominent issue. This demonstrates the need to design mental health interventions for HCPs that ensure confidentiality is maintained.

#### Time

Lack of time was raised as a common barrier to mental health HSB [38], particularly given that mental health support such as counselling and cognitive behavioural therapy require numerous sessions to be effective [39, 40]. Many studies identified limited time as a barrier and the most prevalent barrier in some studies [41–44]. Teo et al. [44] found that 50% of surveyed allied HCPs said time constraints were an issue. However, a low response rate (292%) was noted in this study, which may have introduced a non-response bias. Gold et al. [45] reported similar findings in 2106 female physicians, with 52% agreeing that a lack of time was a barrier to seeking psychological help. However, only women from a mothers’ Facebook group were surveyed; and so, they may be more likely to face time pressure due to family commitments.

Lack of time as a barrier is also supported by qualitative investigations such as Spiers et al.’s [32] study, which found that locum and part-time doctors had more control over their daily schedules and consequently could maintain better mental health. They found that taking time off work resulted in guilt-induced presenteeism (the feeling of letting colleagues and patients down due to limited staff). These findings demonstrate how time is likely the most common barrier amongst HCPs [35]. This is due to HCPs being expected to work longer and more unsociable hours, often above what they are contractually obliged [46]. Additionally, mental health interventions require compliance and commitment to be effective. Coupled with the time constraint HCPs face, a lack of time becomes a significant barrier.

#### Accessibility

Accessibility, as defined by the World Health Organization (WHO), can be separated into three main aspects: physical accessibility, affordability and information accessibility [47]. Physical accessibility has been divided further here into availability and awareness which was found to be a barrier in addition to affordability.

#### Availability and awareness

Baldwin et al. [48] found awareness of services as a barrier; few junior doctors were aware of the role of Occupational Health. Limitations of this study include its small sample size and age, given the recent introduction of telemedicine and digital health to aid in mental health treatments [49]. The prevalence of availability as a barrier has been shown to be lower in more recent studies by Teo et al. [44] and Gold et al. [45], which cited that 25% and 12% of respondents respectively found that availability of services was a barrier to mental health HSB.

#### Affordability

Cost was found to be a less prevalent barrier and was only mentioned in non-UK studies [35, 44, 45]. Teo et al. [44] found that 35% of respondents stated cost as a barrier to seeking psychological help in Singaporean allied HCPs. Edwards & Crisp [35] also found that 75% stated cost as a barrier. However, the small sample size of these studies (328 & 98) limits their generalisability. Other studies in this review did not ask participants if cost was a barrier, therefore it may be underreported but should be still considered as a possible barrier.

#### Stigma

Stigma refers to negative views assigned to a group of people when their attributes are considered to differ from the societal norms [50]. The identified articles showed that stigma was the most prevalent concern of HCPs for avoiding mental health treatment [37, 45].

The stigmatised attitudes towards mental health influence HCPs’ decisions to seek help for mental health [34]. This has resulted in HCPs viewing mental health diagnoses and subsequent treatment as embarrassing, shameful [41, 45] and in some cases a sign of weakness [34–36, 41, 51]. Interviews and surveys of HCPs confirmed that being perceived as weak prevented them seeking help [35, 36, 51].

The internalisation of perceived stigma has resulted in HCPs holding self-stigmatising views on seeking help, demonstrated by Lee, Jeong & Yi [52], who found self-stigma to be strongly associated with attitudes towards psychiatric help amongst nurses. A comparative study between military doctors and personnel showed self-stigmatisation to be more prevalent within the medical profession. This comparison may not be transferrable to doctors outside of the military [53].

Spiers et al. [32] found that self-stigmatising views have caused doctors to believe mental illness diagnoses represent a failure in their roles as caregivers. The presence of self-stigma causes HCPs to hold themselves to a high standard in the eyes of the public. They may also fear that a mental health diagnosis would contradict the healthy image expected of them.

#### Negative career implications

The fear of repercussions on their career was a barrier commonly found within the articles [32–34, 36, 45, 51, 54].

Rees et al. [36] stated fear of repercussions as a reason for refusing to disclose mental health illness to others. This is supported by Bianchi, Bhattacharyya and Meakin [51], who found participants were specifically fearful of being struck off the medical register and suffering a loss of income. The true extent of the problem is unknown due to the small sample size of 12 consultants using non-anonymised interviews. Negative career implications were found to be a key factor in the avoidance of treatment altogether [37]. This was supported by West et al. [54], who surveyed 5829 physicians. 40% stated reluctance to seeking formal medical care due to the impact this could have on their medical license. Adams et al. [34] specifically found that career progression was a much stronger issue for women and those with a history of depression, showing a need to address this concern in these groups with future interventions.

Fears about career implications may be more noticeable among HCPs due to pressures identified in university. Worley [55] identified that medical students experiencing mental distress were discouraged from seeking help or disclosing illness, due to fears of impacting progression.

Axisa et al. [41] and Edwards & Crisp [35] found alternative views in their respective studies. Axisa et al. [41] surveyed 67 physician trainees in Australia and found only 27% were concerned about disclosure of their mental health harming their job applications [56]. This is in contrast to previous studies, however, may be due to its small sample size and differences between healthcare systems as BMA mental health disclosure regulations do not exist in the Medicare model in Australia [56].

#### Negative evaluation of therapy

There was a negative evaluation of therapy amongst the participants of some studies [32, 36, 44].

Spiers et al. [32] found that doctors felt treatment would be unhelpful, describing mental health services as not ‘suited to them’. They also explained the difficulty in switching from a doctor to ‘patient role’. A study by Teo et al. [44] stated that nurses experienced negative perceptions of therapy more frequently than the public due to existing attitudes within the workplace culture regarding mental health conditions.The study also explored the role of country specific cultures, stating attitudes towards mental illness in Singapore resulted in even more negative views of therapy, thereby limiting the generalisation to other countries.

#### Preventing actions

Many articles explored the alternatives to individuals disclosing a mental health condition or seeking help. Schwenk et al. [37] found that 39 physicians in their study (30%) prescribed their own antidepressant medication. Informing others of the individual’s mental health condition became problematic once the individual self-medicated, due to potential fitness to practice concerns, thereby, reducing HSB. Adams et al. [34] found 76.4% of those who self-prescribed would be more reluctant to involve an external party in their illness. This study also stated that those doctors would rather treat themselves rather than visiting a professional. Van der Bijl & Oosthuizen [57] notes that only 15% of physicians in their study visited a GP within the previous year, and further stated that only 6% would disclose their condition to a colleague before self-treating. Gold et al. [45] supported this through a study of 288 nurses, who stated they would not disclose a mental health illness or seeking treatment. 274 would go even further to writing their own prescription or asking a friend to do so.. The study only involved female participants which may have skewed the results due to behavioural differences between genders.

Instead of seeking treatment, HCPs take matters into their own hands to resolve the problem. Bianchi, Bhattacharyya & Meakin [51] reported doctors would carry out maladaptive alternatives instead of disclosing their mental health condition, such as alcohol abuse. This was to the extent that the doctor would not seek help until they suffered a breakdown, or an acute admission to a mental health unit.

### Facilitators to help-seeking behaviour for mental health

#### Workplace culture

Spiers et al. found that doctors who openly speak about their mental health issues also encourage their colleagues to seek support. The study further highlighted that emotional and practical support from colleagues can help doctors in distress seek help. Senior hospital doctors highlighted that a supportive environment stems from good organisational infrastructure with sufficient resources and effective management. These viewpoints were concluded from interviews of a small sample size and therefore, due to the differing culture between HCPs, limits the generalisability of the findings.

#### Support services

GPs found that specialist services were beneficial in supporting their mental health issues, due to the provider’s experience in dealing with doctors and treating them as patients [32]. The confidential nature of these specialist services eased help-seeking [32]. This was supported by doctors in hospitals who favoured using external services that were independent of theirorganisation to ensure their privacy was preserved [51]. Data collection by White et al. [33] substantiated this, with 46% of psychiatrists favouring a local private facility for inpatient treatment compared to 4% choosing a local NHS facility; confidentiality was a deciding factor for most of these doctors. Considering these findings were across three studies, we can conclude that confidentiality of services is of importance to HCPs when seeking mental health support.

#### Summary of the SLR

This SLR identified nine barriers and two facilitators. From conducting this review, we have found that there is a lack of research identifying the facilitators to seeking help for mental health. Consequently, further research is required to uncover additional facilitators.

## Qualitative data collection

### Methodology

#### Study design

The primary data collection will consist of interviews (see Additional File 3 for the interview guide) and leads on from the SLR in order to develop the barriers identified, as well as to specifically increase information on previously unexplored facilitators to mental health HSB. The rationale for these methods is summarised below.

#### Choice of sampling method

Participants were recruited for interviews using a self-selection method. This acknowledges that the interviewees opting in may be more comfortable to speak about sensitive topics [58]. Researchers Khadeejah Mujahid (female) and Fahmid Ahmed (male) conducted the interviews. No relationship was established between the interviewees and interviewers prior to the interview.

#### Sampling process

The sampling was undertaken using the pre-determined inclusion and exclusion criteria (Table 3). Initially, all HCPs were to be included in the study as healthcare staff are at increased risk of experiencing mental health issues, especially given the COVID-19 pandemic [59]. However due to resource and time constraints this was not feasible within the scope of our study. Only NHS doctors were recruited to control for cultural differences across different countries as this has been shown to impact aspects of mental health including perceptions and treatment seeking patterns [60]. Thirty-two respondents expressed interest, while 31 were eventually recruited.

**Table 3:** Criteria for participant recruitment

Inclusion Criteria	Exclusion Criteria
Doctors	Other HCPs
Doctors who have worked in the NHS	Doctors who have no experience in the NHS
Age: over 18	Age: under 18

Participants were recruited using three main channels: social media, personal contacts and through organisations, such as the Royal Colleges. Once they had agreed to be interviewed, participants were sent the Participation Information Sheet (PIS) (Additional file 5) and Consent Form to be completed before the interview.

#### Data collection

This SLR has revealed a gap in the literature:

1. There were a limited number of articles focused on finding facilitators to mental health HSB for HCPs.

Our search terms were comprehensive, which meant that any articles relating to facilitators should have arisen. The lack of articles on these topics allows us to conclude that these are two areas which have not previously been researched. To address these, further research is required. Despite HCPs being the focus of the SLR, most of the articles discussed the barriers and facilitators faced by doctors. Therefore, the barriers and facilitators to HSB found from these studies may not be generalisable to those faced by all HCPs.

#### Participants

Although our search terms encompassed HCPs, the majority articles selected focused on doctors. As a result, our primary data collection will focus on doctors for the following reasons:

1. Focusing on a subpopulation increases the likelihood of yielding representative data.
2. There is a shared commonality between doctors in their training. Irrespective of their speciality, all doctors undergo a similar 6-year medical school programme. Research shows that negative attitudes to seeking help stem from early stages of medical training [61, 62].
3. Doctors are exposed to similar determinants of HSB such as culture and stigma.

#### Design of data collection methods

The online video conferencing platform, Zoom, was used for interviews to allow for greater time flexibility due to the elimination of need to travel to a common space [63]. It also allowed participants to be comfortable and safe [64]. The interview questions were based around the barriers and facilitators identified in the SLR.

#### Thematic analysis

Thematic analysis was chosen to analyse the interview data as it has been shown to be effective in identifying, analysing and reporting patterns in qualitative data. This would aid us to identify novel barriers and facilitators to HSB in doctors. It is also beneficial in highlighting similarities and differences in the data, which would help generate codes to assign to the themes [65]. Three researchers encoded the data. Data collected was processed through Microsoft. COREQ guidelines were used for the qualitative research.

The interview data was analysed according to Braun & Clarke’s (2006) six phases of thematic analysis. These phases of thematic analysis are as follows:

1. Familiarise yourself with the data.
2. Generate initial codes.
3. Search for themes.
4. Review the themes.
5. Define and name the themes.
6. Produce the report.

Phase 1 involved all researchers transcribing the interviews using Otter AI, an AI-powered transcriber and then checking each transcript manually. Phase 2 involved generating the initial codes, where the data was categorised into phrases with similar meanings. This was trialled with four interviews first to ensure agreement the coders on the level of detail and format of the codes. The initial codes created were for the entire data set and they were compared to identify any areas of similarity so codes could be combined. An example of the codes generated can be found in Table 4.

**Table 4:** Example of coding the interviews

Interview transcript extract	Codes generated
I think, to be honest time perhaps, because like there’s not an easy way to seek it. Or at least I used to think that was the case for instance like I thought you had to like make a GP appointment, and then you’d like get referred through that process rather than you know just like googling stuff and then just seeking mental health that way	1. Time as a potential barrier to seeking help 2. Accessibility as a potential ba rrier to seeking help 3. Online access is easier

Phase 3 involved sorting initial codes into themes. We recognised that themes could be organised into different levels, so we arranged them into ‘meta-themes’ which encompassed barriers and facilitators. Each barrier and facilitator were labelled as a ‘sub-theme’.

In Phase 4, the themes were reviewed at two levels; firstly, at the level of the coded extracts to ensure themes fit a clear pattern throughout and secondly, across the entire dataset. This dataset was checked again to identify any new themes. In Phase 5 the themes were defined and named, to ensure they were understandable. Finally, Phase 6 involved generating the report. Excel was used to create a filtered table of all the quotes so sufficient evidence could be easily found for each theme, to create an analysis of both the barriers and facilitators to HSB as shown in Chapter 5.4.2.

## Results

Results of the semi-structured interviews are presented in this chapter. The following meta-themes were identified from the qualitative analysis:

- Perceptions about mental health
- Confidentiality
- Career implications
- Time
- Accessibility
- Culture
- Preventative factors

**Fig. 3:**
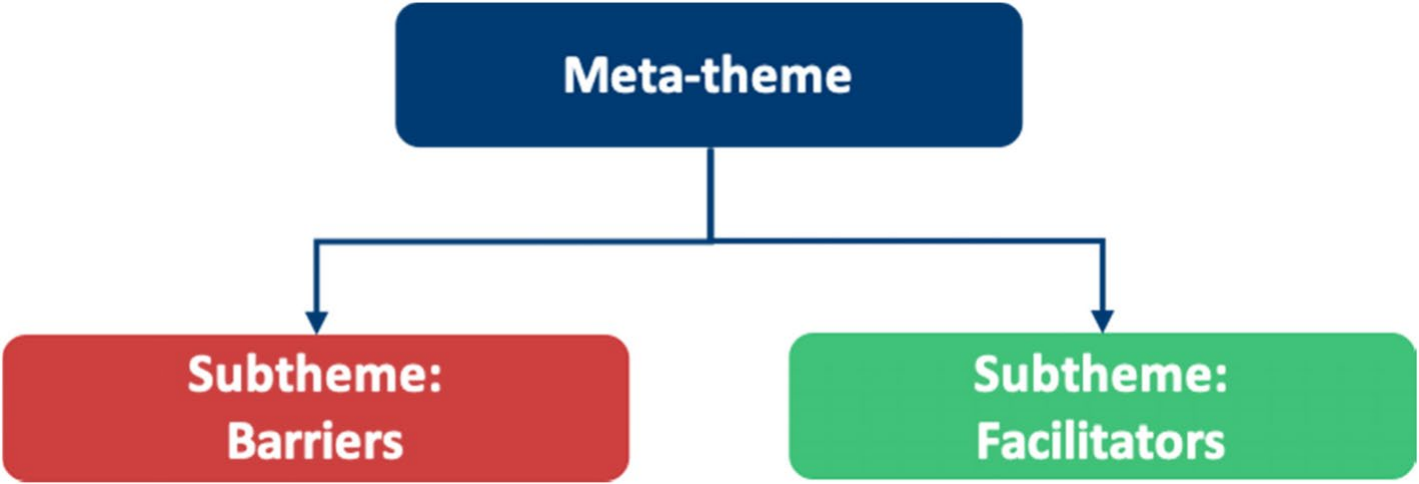
Meta-themes encompass barriers and facilitators

The barrier and facilitator aspects of these meta-themes (as shown in Fig. 3) will be discussed in this chapter.

### Research results and meta-themes

Our qualitative data aimed to investigate the barriers and facilitators of HSB for mental health in 31 NHS doctors (see Table 5).

**Table 5:** Interviewee demographics

	Number of Participants	Percentage (%)
Gender		
Male	16	52
Female	15	48
Age		
20 to 34	18	61
35 to 44	9	29
45 to 54	2	7
55 to 64	1	3
65 and over	0	0

### Overview of meta-themes

**Fig. 4:**
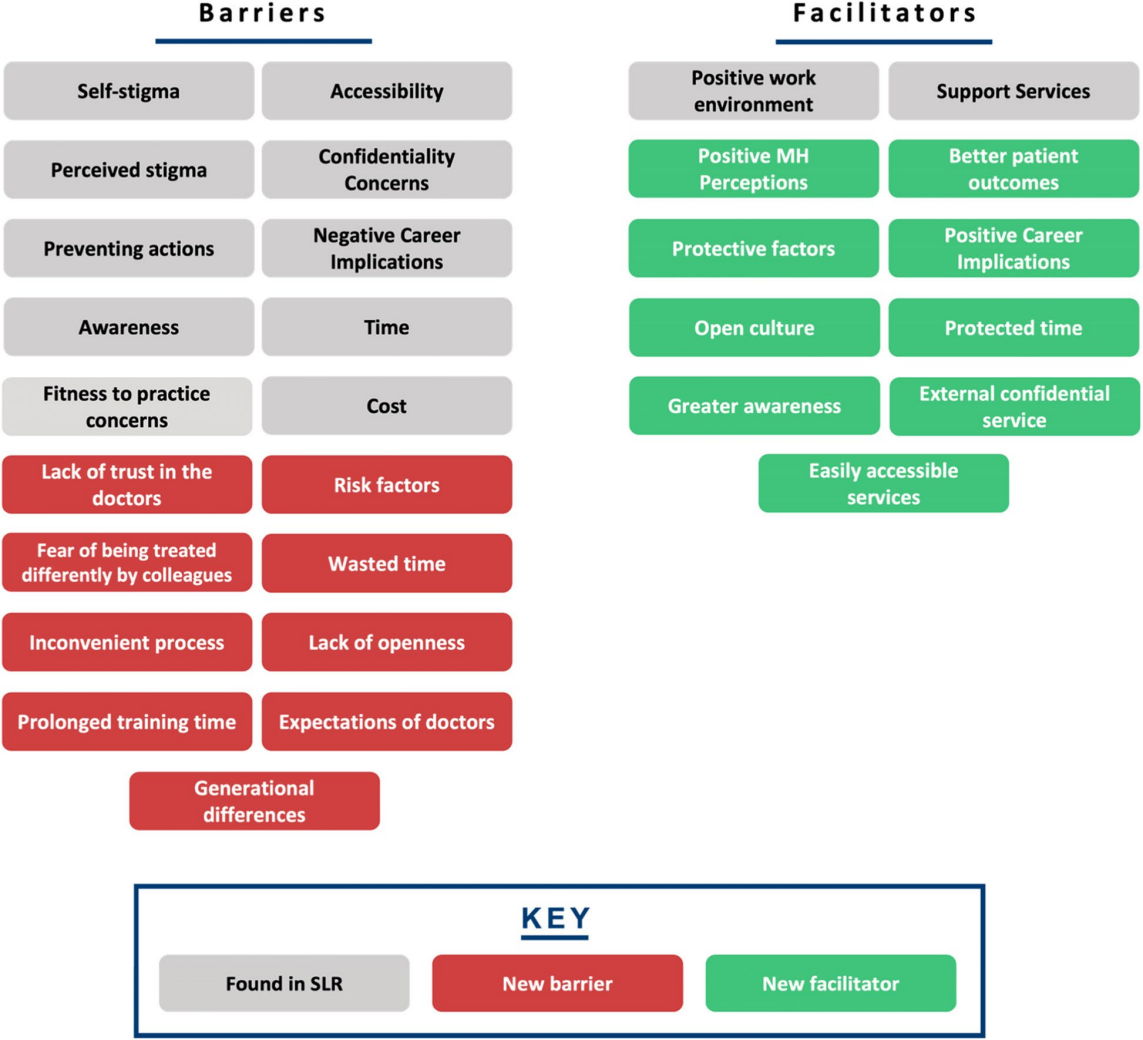
Additional subthemes identified by qualitative analysis

Our research is summarised into themes shown in Fig. 4. This figure highlights the additional facilitators to HSB in doctors not found in our SLR, fulfilling our objective to broaden the research into facilitators where barriers had been explored previously. Table 6 highlights the breakdown of each meta-theme into its barrier and facilitator components.

**Table 6:** A breakdown of meta-themes into their barrier and facilitator components

Meta-theme	Barrier	Facilitator
Meta-theme 1: Perceptions about mental health: refers to the views an individual holds about mental health	The negative views fall under perceived and self- stigma	The positive views under positive perceptions about mental health, as shown in Fig. 5
Meta-theme 2: Confidentiality concerns are worries regarding anonymity when seeking mental health support	Doctors worrying that they will know the HCPs who will be treating them in a professional capacity, leading to a lack of divide between personal and professional life	Having a confidential service for doctors that is separate from their workplace, as shown in Fig. 6
Meta-theme 3: Perceptions of career implications include ideas or concerns about the impact of seeking help on future career prospects	As a barrier, this includes prolonged training periods due to fears of being seen as incompetent	As a facilitator, this includes being able to provide better care for patients through seeking help for oneself when needed, as shown in Fig. 7
Meta-theme 4: A common theme uncovered within the interviews was the concept of time	Time acted mostly as a barrier to seeking help	Time acted as a facilitator to seeking help in a limited capacity, as shown in Fig. 8
Meta-Theme 5: Awareness and Accessibility A lack of awareness of mental health services and accessibility issues are factors which demote the use of mental health support by reducing the availability	Greater awareness and easy accessibility for doctors provided increased encouragement to seeking help	A lack of awareness meant even doctors willing to seek help are unable to. Poor accessibility to these services acts as a deterrence and could lead to worsening of mental health (Fig. 9)
Meta-theme 6: Culture is defined as “the ideas, customs and social behaviour of a particular people or society” [62]. Culture can be categorised into organisational and societal culture where organisational culture in a healthcare context “represents the shared ways of thinking, feeling and behaving in healthcare organisations” [62]. These may include multiple subcultures and comprise three levels: visible manifestations, shared ways of thinking and deeper shared assumptions [66]	The barriers fall under structural stigma identified in the SLR which refers to “societal-level conditions, cultural norms, and institutional policies that constrain the opportunities, resources, and wellbeing of the stigmatized” [67]	Facilitators include open culture and supportive seniors, as shown in Fig. 10
Meta-Theme 7: Preventative Factors Preventative factors are defined as any factors that could prevent a doctor from seeking professional help in the first instance	There are ‘protective factors’ which decreases the chance of a negative mental health outcome	There are ‘risk factors’ which may increase the chance of a negative mental health outcome, as shown in Fig. 11

**Fig. 5:**
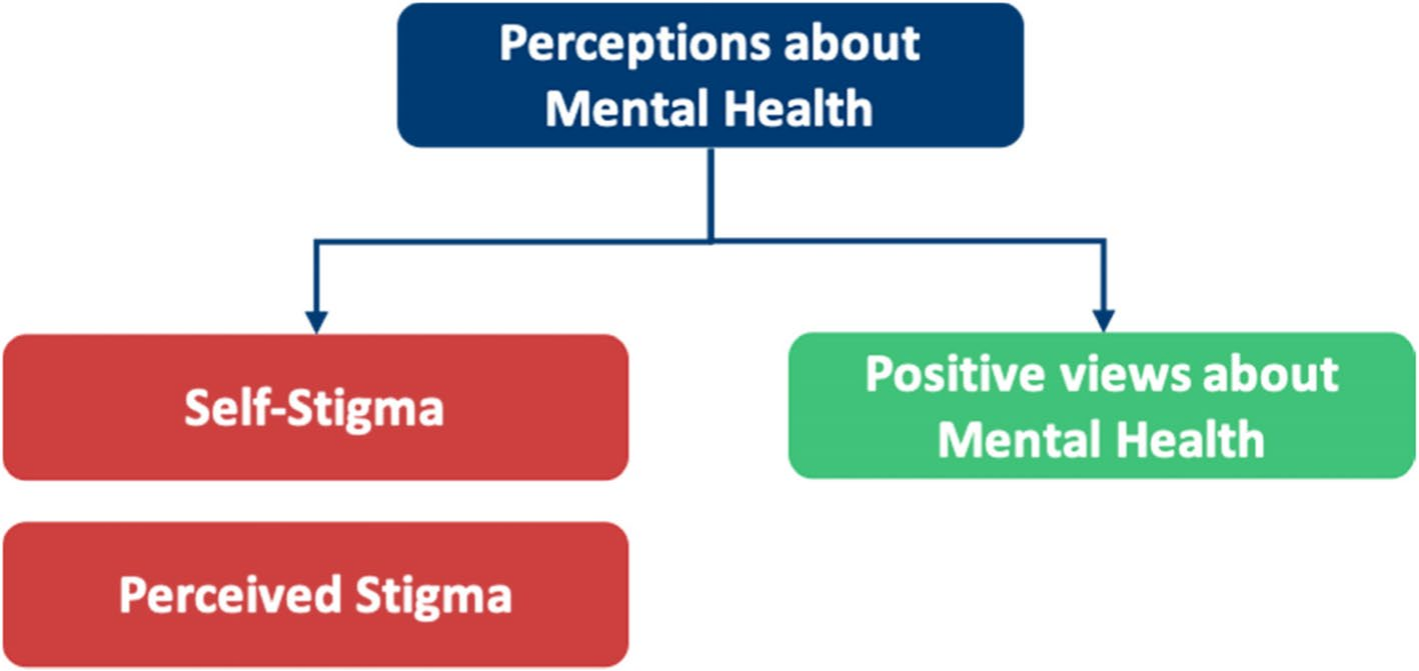
Barriers and facilitators for ‘Perceptions of Mental Health’

**Fig. 6:**
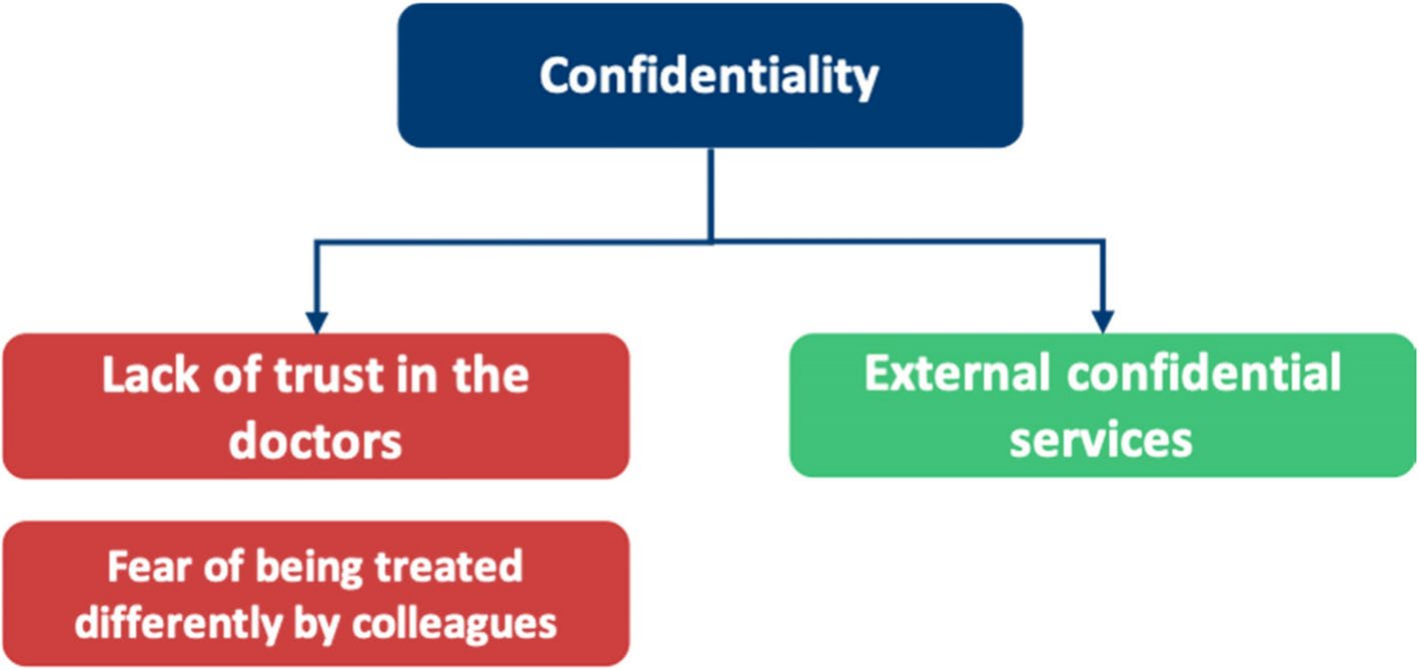
Barriers and facilitators for ‘Confidentiality’

**Fig. 7:**
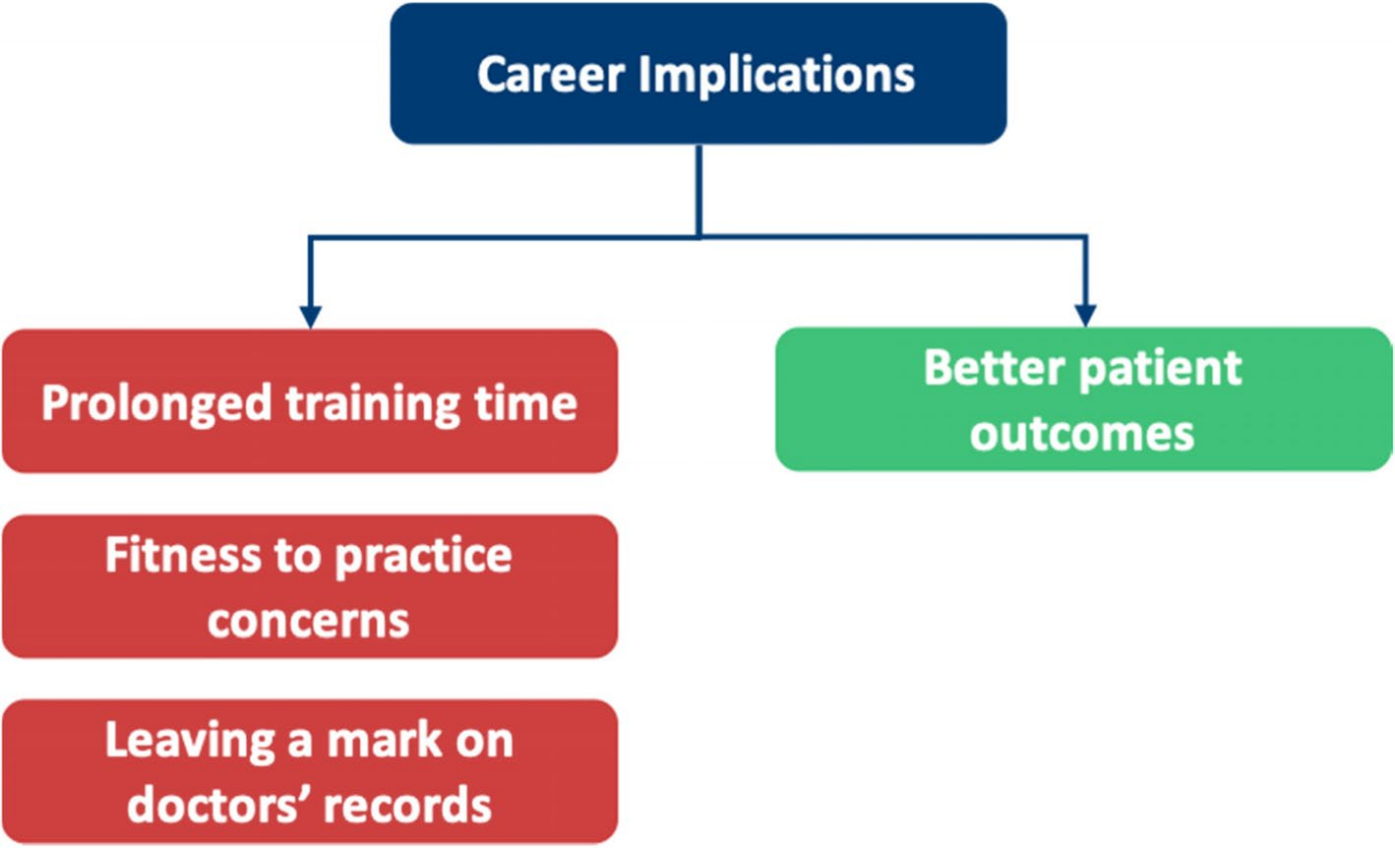
Barriers and facilitators for ‘Career Implications’

**Fig. 8:**
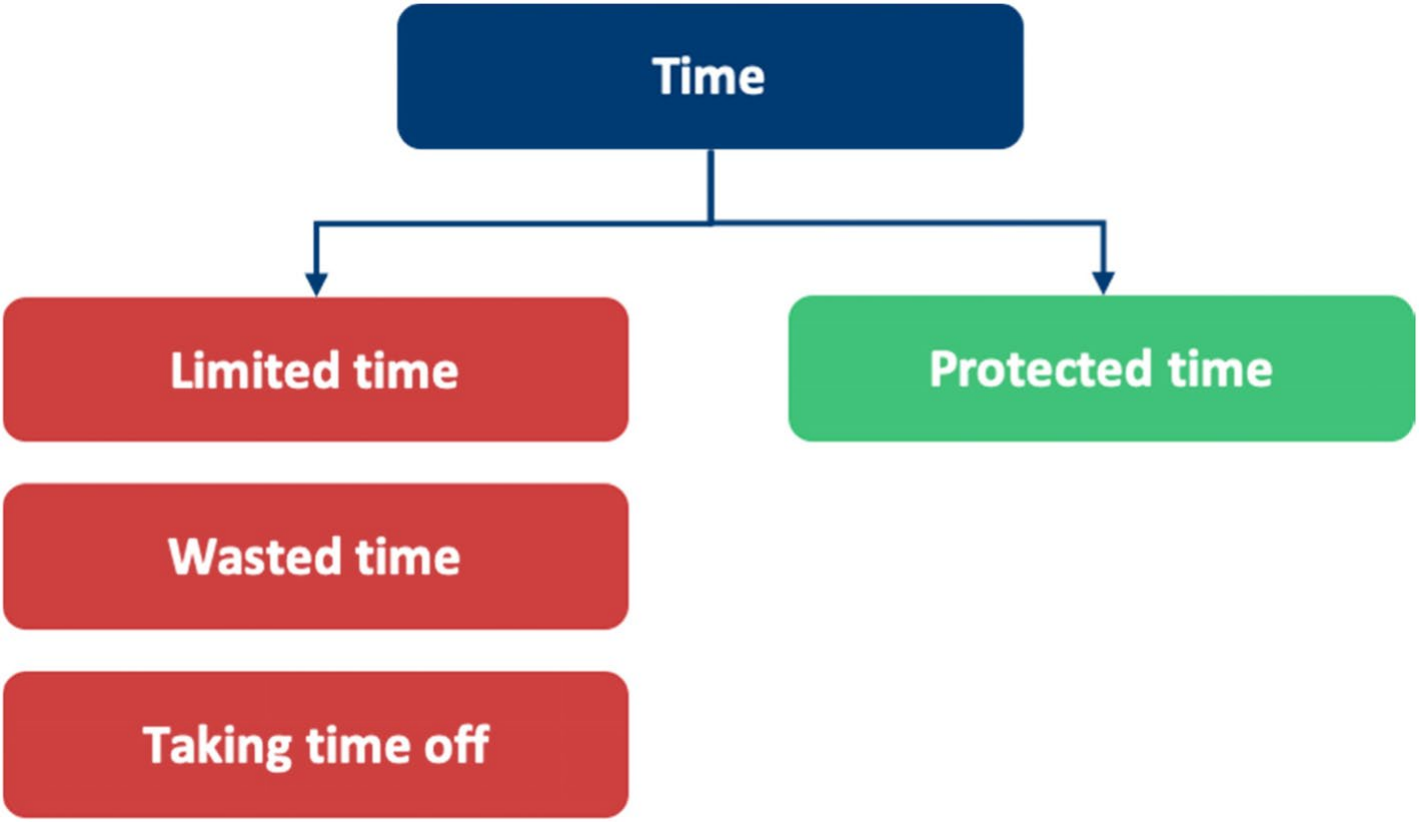
Barriers and facilitators for ‘Time’

**Fig. 9:**
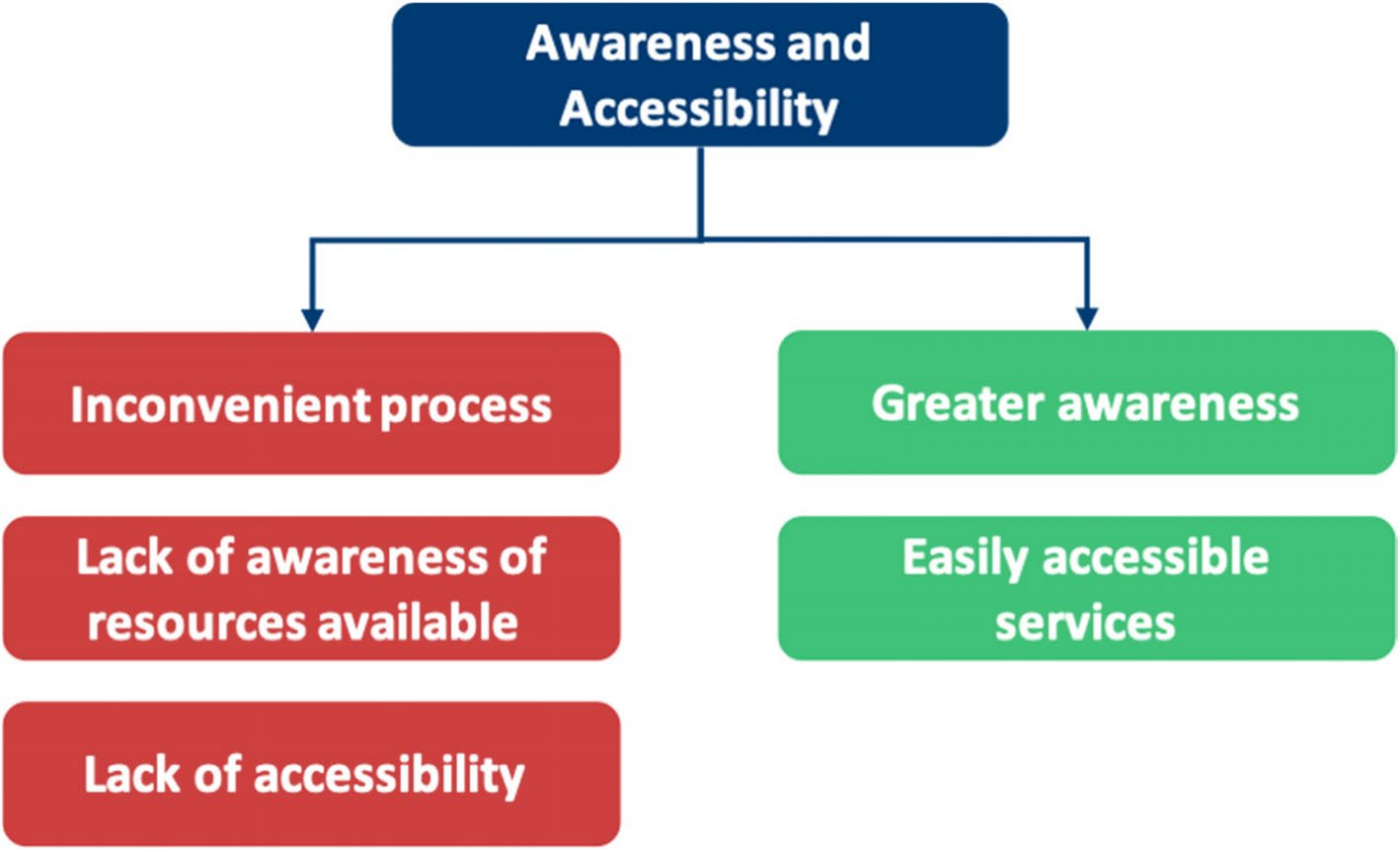
Barriers and facilitators for ‘Awareness and Accessibility’

**Fig. 10:**
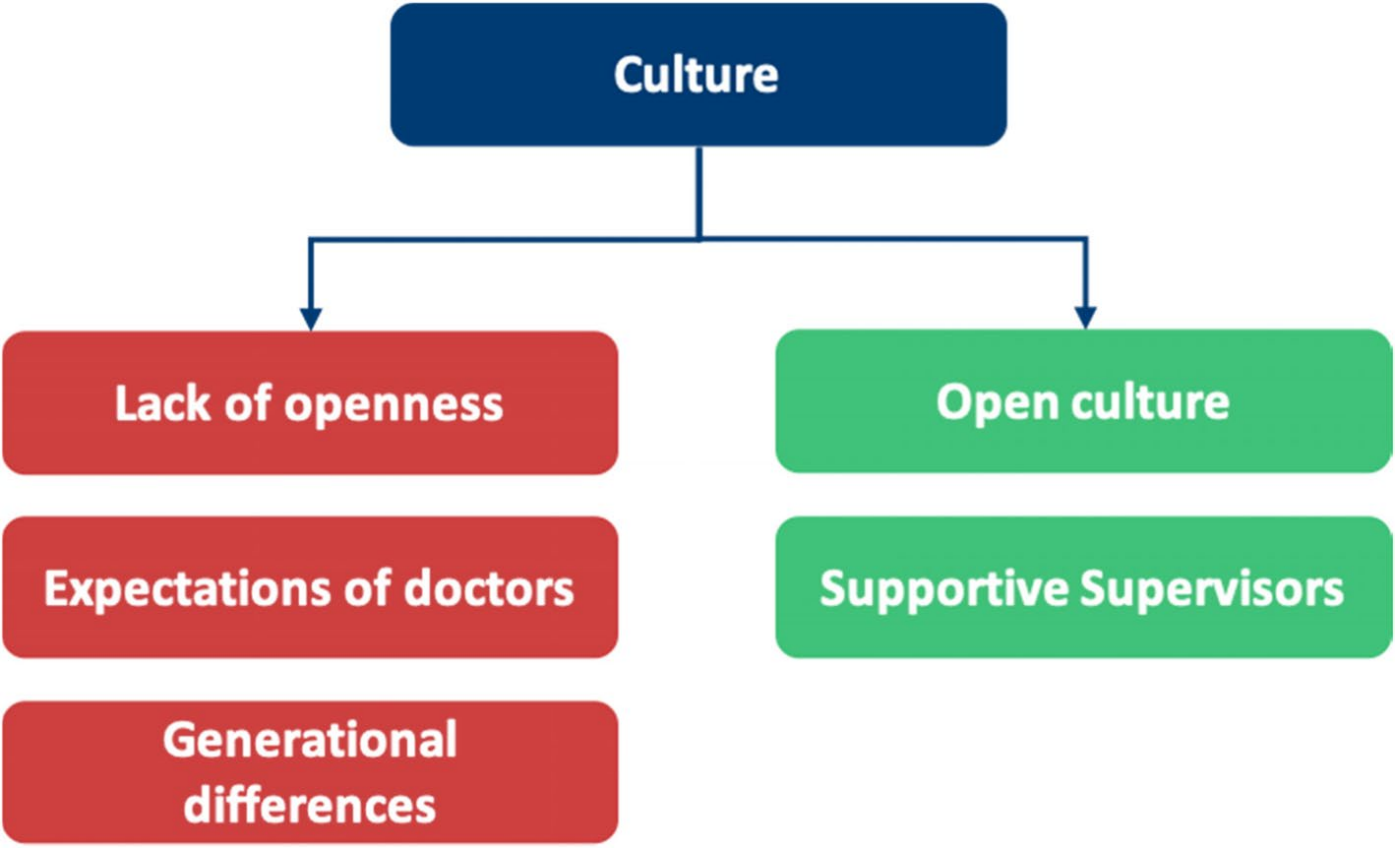
Barriers and facilitators for ‘Culture’

**Fig. 11:**
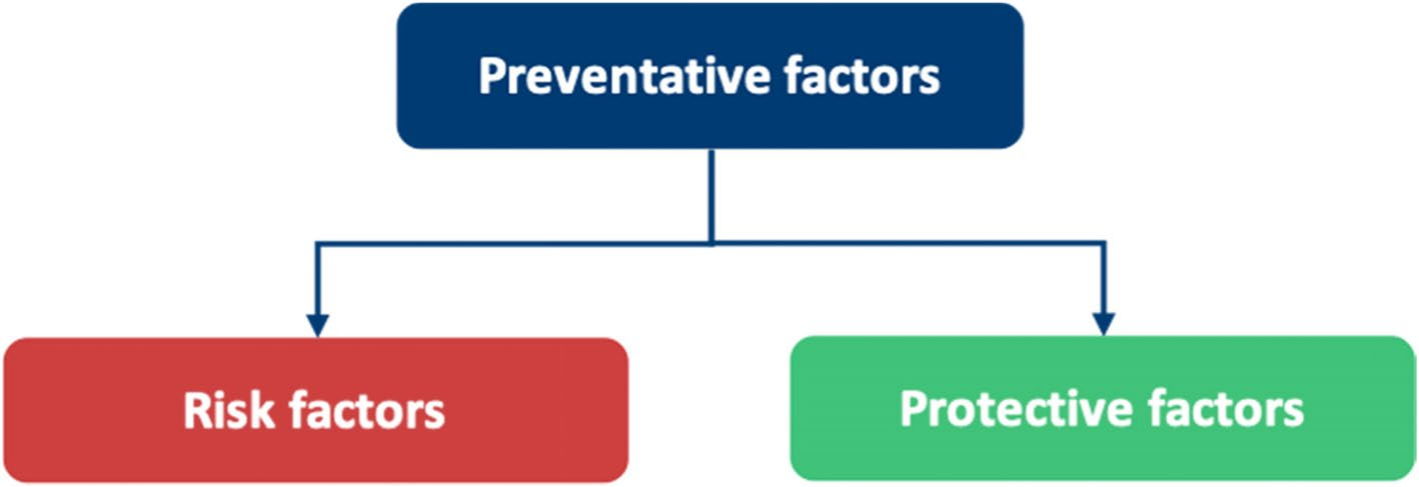
Barriers and facilitators for ‘Preventative Factors’

## Meta-Theme 1: Perceptions about mental health

### Perceptions about mental health as a barrier

#### Perceived stigma

Perceived stigma is the fear of being discriminated against, as outlined in our SLR. Our qualitative analysis found this to be the most prevalent barrier cited by doctors. Many doctors were concerned about being perceived as weak by their colleagues and this prevented them from fulfilling their work responsibilities.

‘[Your seniors] would see that you weren’t coping, and so they would probably give you less work or expect less of you.’ (I1)

‘[There is the view that] you should deal with that stuff in your own time… it shouldn't affect your work’ (I7)

These quotes describe the perception that doctors feel they may be seen as weak for seeking help; this was often linked to the expectation that they should be invincible and not allow personal issues to impact their work. Whilst these interviewees described different treatment from colleagues and seniors, some interviewees felt that they would be deterred from seeking help due to the indifference towards mental health from their senior colleagues.

‘[Supervisors] actually don’t really care. They’re the type of educational supervisors who are just like… ‘We’ll just sign off, whatever, we’ll have this meeting and do it as a formality’, but they don’t actually look into how you’re doing.’ (I6).

‘I don’t know many people in senior positions that openly talk about mental health or care about it.’ (I16)

Compared to quotes from I1 and I7, the above quotes show that doctors may fear the perceived stigma of mental health, stating the ease of signing forms and navigating bureaucracy, rather than engaging in conversation about difficult subject matters.

#### Self-stigma

Self-stigma is the internalisation of negative societal views about a person with a mental health diagnosis and conforming to stereotypes [61, 68]. In our results, this presented as doctors seeing themselves as weak and feeling ashamed for seeking help for their mental health.

‘It’s almost like… stigma on myself… if I start seeking help or going into that territory, then there’s a label put on it.’ (I6)

‘You are seen as a bit weak and not part of the team… not pulling your weight, you don’t want to be letting the team down’ (I16)

These quotes represent the fear that doctors have of being given a mental health diagnosis or perceiving themselves as weak. Compared to the previous theme, which represented a perceived stigma, this theme presents an internalised fear of how doctors may perceive themselves for seeking help and feel weaker as a result.

#### Perceptions about mental health as a facilitator

These perceptions were either positive views about others who sought help or positive views held by the interviewees about themselves seeking help. The latter was less prevalent, emphasising how most interviewees thought they would be seen negatively for seeking help yet would see colleagues seeking help in a more positive light.

If someone were to confide in me, and… tell me they were struggling… I would see it as a sign of maturity.’ (I7)

‘I would actually probably admire, commend them for actually going through that process.’ (I8)

This theme shows an interesting juxtaposition of the fear of perceived stigma, compared to how doctors would view others seeking help as a sign of’maturity’ or bravery. This represents the importance of honest and open dialogue surrounding mental health HSB in the medical workplace and culture.

## Meta-Theme 2: Confidentiality

### Confidentiality as a barrier

#### Lack of trust in the doctor treating them

Some interviewees raised the concern of being unable to trust the doctor treating them with regards to confidentiality.

‘I just don’t like my GP very much. He's not a bad doctor. He’s a really good doctor, but he’s just like an old man… he knows my whole family… he’s known me since I was 15… I’m a very different person to that person. So, I just find it really awkward to talk with him.’ (I14).

‘You potentially know your colleagues… that’s a barrier because you feel as though they might know someone you know, and although we’re all very professional… you’re a bit worried about confidentiality.’ (I15).

These quotes show that some doctors would avoid bringing up mental health issues to their own doctors in the same way that they would bring up a physical issue. Quite often this was due to knowing the doctor too well or not having a sufficient rapport with them. Some of the doctors interviewed want a doctor they can trust, but it should be someone they do not have a prior personal relationship with.

#### Fear of being treated differently by colleagues

Interviewees feared that colleagues finding out that they had sought help for their mental health could result in colleagues losing respect for them and treating them differently.

‘If the confidentiality is maintained… that person doesn’t lose esteem with his colleagues.’ (I26)

‘Confidentiality is important, so that person does not have that variable treatment by different people.’ (I26)

The doctor above viewed broken confidentiality as something which could lead to’lost esteem’ among colleagues, with mental health HSB potentially being something negative if it was discovered by workplace colleagues or seniors.

### Confidentiality as a facilitator

#### Confidential service external to workplace

A few interviewees mentioned that when seeking help for their mental health, they would want to go to a confidential independent service, to maintain a divide between their personal and professional lives.

‘I’d want to go to someone completely independent that I didn’t know and didn’t have any access to my work colleagues… completely confidential and separate.’ (I1).

‘That’s [confidential services] where the Practitioner health programme is very helpful.’(I4)

Doctors appreciated having confidential services available to them through Health Education England and the NHS Practitioner Health Programme; they felt empowered to seek help.

## Meta-Theme 3: Career implications

### Perceptions about career implications as a barrier

#### Prolonged training

Career implications was identified by nineteen the interviewees as influencing likelihood to seek help. One barrier identified was the concern that seeking help would prevent doctors from reaching the expected standard to progress in their career:

‘Instead of what I would expect it to be if you’re having a rough time – ‘… take time out whatever’, it was tied up as, 'You haven't met the expected standard… you need to repeat posts, you need to spend more time in training because you haven’t hit the required standards.’ (I2).

‘It can make it [feel] like you’re training [for] really long, or you can fail some aspects… that can be really traumatising.’ (I14)

A medical career is associated with a long training programme and the doctors highlighted the fear of seeking mental health help leading to a further extended programme because of needing to take time out.

#### Leaving a mark on doctors’ records

Another subtheme identified was the potential mark that seeking help would leave on a doctor’s reputation.

‘You stay in the programme full time, or you leave, and then you lose your training contract and that’s a big black mark in your career…. encouraging flexibility and allowing people to do what works for them would be hugely beneficial.’ (I2).

‘My life insurance policy doesn’t allow for mental health issues to be a reason why I’d claim for that, because I was incorrectly labelled as depressed when I was 20-something coming out of uni.’ (I18).

Doctors were concerned that previous mental health issues would be picked up on by future employers and workplaces. Interviewees felt that the impact of this mark on their record outweighed the benefit of seeking help.

#### Fitness to practice concerns

The final subtheme encompasses fitness to practice concerns. Doctors felt that seeking help showed they were unable to cope with the demands of their job and formal disclosure would result in negative career implications.

‘There’s perhaps a fear that maybe if you do disclose any struggles that you’re having that it might be that deemed by are, you know, not competent at your job, or this is affecting your work.’ (I21).

‘There’s the assumption that if you're not coping, well, mentally, or whatever, then you’re not fit to be in work or… looking after patients.’ (I30)

The fear of appearing incompetent due to mental health issues was cited. Doctors felt it would be better to show that they were coping well with their jobs, rather than to disclose any struggles, to maintain the expectation that a doctor’s fitness to practice includes a flawless mental health record. Career Implications as a Facilitator.

#### Better patient outcomes

Career implications were seen to enhance the likelihood of seeking help, as interviewees felt that doing so would result in better patient outcomes.

‘We have a responsibility, if we’re struggling, to get help because the GMC are very much like, you need to see yourselves… you have responsibilities to look after yourselves. And if you don’t look after yourself then you won’t be a very good doctor.’ (I7).

‘It is better for you to seek the help that you need and it’s better in the long term also for your clinical practice or for your patients as well.’ (I11)

Seeking help was seen as adding value to a doctor’s ability to deliver care to their patients, with the perception that caring for themselves would improve their ability to take care of others.

## Meta-Theme 4: Time

### Time as a barrier

#### Limited time

Many doctors identified that time was limited in their profession; this often meant they would not have enough time to either seek help externally or attend any sessions at work.

‘There’s just not enough time to go through that process [finding mental health services].’ (I8)

‘We don’t have that much time… you have to go to work and then you also have stuff outside of work that you have to do, so that’s the only thing that would put me off to be honest with you… the time and the commitment issues.’ (I9).

Evidently, many interviewees worried that if they needed help, they would be unable to access it due to a lack of time, due to their busy working schedules.

#### Taking time off

Interviewees shared the difficulties in taking time off to seek mental health support. This commonly presented as worry about being unable to fulfil their duties towards their patients and fear of increasing the workload of their colleagues.

‘One thing that came to mind was what my grandfather would say to me, you need to go to work, you’ve got patients to look after… as doctors, we have a responsibility to look after other people, and it’s that sense of what might happen if I don't go in.’ (I19).

‘If it’s an on-call, it’s a real nightmare to just randomly call in sick, because then that needs to be covered at the last minute by someone else… it’s a lot easier to plan it in advance as annual leave or study leave… all of us have to think at least two or three times before we call in [sick] because I think compared to a lot of other professions, the consequences are more troubling.’ (I19).

These doctors cited the fear of abandoning their patients as a result of taking time off. Due to the nature of the role of a doctor, the best interests of patients often takes precedence over the health of doctors themselves.

#### Wasting time

Seeking help was referred to as a waste of time by some doctors. This idea was linked to the long waiting times for referrals and the effectiveness of those services.

‘The duration of how long you get with somebody would definitely need a limit… like counselling therapy. If I get for a short amount of time I’ll definitely be quite wary to try it… it’s a lot of investment of effort and time to even think about calling.’ (I3).

‘I think it’s that self-limiting belief of am I just going to end up wasting more time trying to fix this by seeking these resources out?’ (I10)

These doctors discuss how time is an investment and one which they would be unwilling to take if the effort outweighed the value of seeking help. The doctors mentioned how they would benefit from a long-term solution.

### Time as a facilitator

#### Protected time

The only facilitator mentioned was the idea of protected time, defined as a certain time in which normal duties of a doctor are not expected.

‘Would be really good if [Balint Groups were] worked into every session, I don’t know where the time is for that unfortunately, but… a lot of programmes have lots of teaching like lecture-based teaching built into it. And I wonder if maybe some of that could be replaced with like half an hour to an hour of Balint Group type therapy sessions.’ (I3).

‘I think having a space in the workplace… a dedicated time, protected time to be able to discuss any problems or challenges…with colleagues, might help.’ (I21).

The doctors suggest that compulsory discussion time should be introduced, in a similar way to scheduled teaching time in training posts. This would serve as a regular time to discuss and share concerns with others.

## Meta-Theme 5: Awareness and accessibility

### Awareness and accessibility as barriers

#### Inconvenient process

Inconvenient processes discouraged doctors from seeking help. Accessing support services were considered complicated and, ultimately, not worth the time and effort, as it creates further burden and stress.

‘To be honest… there’s not an easy way to seek [help for mental health].’ (I8)

‘[Accessing mental health services is] very complicated… to get a mental health referral someone might have to go through occupational health or go through the GP… There’s so many different pathways that it’s very easy for patients to get lost.’ (I30).

#### Lack of awareness of resources available

Interviewees also mentioned that a lack of knowledge of the available services prevented them from utilising them, expressing the need for more awareness for doctor-specific resources.

‘Who do you speak to? When do you speak to them?’ (I5)

‘Not entirely sure that there's very good provision for doctors who actually suffer from mental illness.’ (I3)

#### Lack of accessibility

The interviewees expressed that the difficulty in accessing mental health services impeded them from utilising the available support. Doctors shared the difficulty of scheduling counselling sessions and the lack of regular sessions or a centralised forum to speak openly dissuaded them from seeking help.

‘Counselling [was] like scheduling the meeting. And that itself created quite a lot of stress.’ (I24)

‘People don’t [seek help] because it’s not… obviously there. I think if it was very clear that it was there all the time that… there was somebody you know, there to do that. And there was a forum to go into, I think it would, the uptake would be much higher.’ (I28).

### Awareness and accessibility as a facilitator

#### Greater awareness

Increased awareness of the mental health services available was highlighted to increase engagement and improve HSB. Some doctors specifically mentioned that being aware of effective services recommended by others would encourage them to use them themselves.

‘If there was a very obvious forum for [mental health available support].’ (I28)

‘Something that I can access myself when I want to, and I can book an appointment that's convenient for me, probably that will be based online as opposed to having to attend, I would find that more convenient and therefore be more likely to access it.’ (I1).

#### Easily accessible services

Accessibility was stated numerous times in reference to mental health services.

“Ease of access self-refer to [mental health services], have access, not needing to go and see a GP and get a GP referral through because that takes time.” (I1).

“If they streamline things, if they made everything more readily available, so from uni onwards.” (I8)

Interviewees often mentioned how the process of access help was long and convoluted; they said a more direct and easily accessible approach would encourage them to seek help.

## Meta-Theme 6: Culture

### Culture as a barrier

#### Lack of openness around mental health

Many doctors stated that a lack of openness around mental health as a result of negative culture was a barrier to seeking help for mental health. Some doctors mentioned that many non-medical fields had strong systems in place to allow employees to seek support for their mental health. It was also cited those processes were designed to support them felt like a formality, rather than providing the emotional support they required.

‘I feel like within doctors themselves, there isn’t much of a culture of talking about mental health, especially from senior doctors.’ (I6)

‘I think it’s this idea that you know, you’re a failure, there’s something wrong with you [if you are diagnosed with a mental health problem], but how can there be something wrong with you when you’re supposed to be in a profession where you’re looking after people? So, I think that really holds people back from admitting to a problem.’ (I24).

Doctors from certain specialities were more likely to face barriers due to differences within subcultures. The interviewees cited surgery as a “classic” example of a speciality which required doctors to focus on their jobs rather than their own health, while some doctors associated general practice as a more supportive speciality and culture.

‘I think surgeons do not come forward a lot. Just because surgery has a bit of, I don’t want to say machoistic, but it does have a bit of a "we are the tough people" attitude.’ (I20).

‘As a GP trainee, it’s nice because especially when you’re doing your GP placement, you [spend] a lot of time with your supervisor [and] you get dedicated… teaching session[s] … compared to your F1 and F2, where your supervisor is kind of just like a name; a name on your forms.’ (I14).

‘And I think that people feel uncomfortable speaking about [mental health, because] they worry about judgement upon them for that. And I think that’s probably the main barrier. I think… if you were to raise these issues, you know, may people judge you? Or could it affect your working relationship with people in your work environment? Could that have a negative impact on your career?’ (I25).

Doctors were also fearful of judgement from their colleagues and seniors due to a lack of openness around mental health. They stated fears of being judged due to workplace culture as a reason for not being open with their mental health, which links to the perceived stigma subtheme identified earlier.

#### Expectations of doctors

Mental health was often perceived by doctors as a vulnerability. This was identified as perceived stigma and was often internalised as self-stigma, as identified in the first meta-theme.

‘If it was like to do with my mental health and how I’m feeling and stuff I don’t really talk about how I’m feeling that much to other people, especially not people at work… maybe it’s just like… it’s not a very professional conversation to have.’ (I14).

‘There’s probably quite a bit of taboo around it to admit that you’re calling in sick for, you know, reasons related to mental health. So, something that I’ve probably noticed is that… sickness is probably underreported.’ (I23).

Due to their fears of judgement and perception that mental health should be kept out of the workplace, doctors stated that related conversations may be seen as unprofessional. Many doctors also saw resilience as a key expectation from themselves, with mental health problems or sickness representing a taboo area.

#### Generational differences

Interviewees stated cultural differences between generations as a barrier to seeking help for their mental health.

‘I think that’s a generational gap partly as well, because like, at least at the hospital I'm at, the age gap is… that [the consultants are] like parent age’ (I8).

‘If we’re not seeing and not having these discussions with seniors… you’re not going to feel safe and comfortable enough… if you can’t even open up to them’ (I6).

These interviewees cited the consultants’ age as a concern for them, comparing this to the age of an older relative, with whom they may not necessarily be able to easy speak about mental health.

### Culture as a facilitator to HSB

#### Open culture

‘Open culture’ was mentioned as a facilitator many times by the interviewees. This included openness within specialities and subcultures and within the NHS as a whole and encompassed open conversation, frequent supervisor meetings and advice from seniors.

‘To have good resources available for doctors to be able to seek… [and] have all consultants, supervisors, people in senior positions, speaking about it, and not making it such a… strange thing [would be helpful] …. There’s Practitioner Health, which people use, but it's quite new. And I don't know anything else that is free and easy for us to access.’ (I16).

‘I think [mental health awareness is] probably changing… maybe… before [it] wasn’t common, but I think now, the majority of doctors’ approach would be open regarding these things.’ (I25).

The interviewees mentioned that mental health issues could be less stigmatised, and services could be made easier to access if people in senior positions were able to have more frank mental health conversations. They also stated culture change over generations as a positive factor which allowed doctors to be more open about their own mental health and encourage the use of support services when need.

#### Supportive supervisors

Interviewees mentioned support from seniors and supervisors as another facilitator for seeking help for their mental health. Supervisors and consultants were identified as key groups who could make doctors feel more comfortable and honest about their mental health. With their senior roles in the workplace hierarchy, these groups were able to encourage junior doctors to seek help, as well be sources of help themselves to ensure that any mental health conditions did not worsen.

‘When I said to my supervisor “I feel really burnt out and really struggling… Is there anything I can do?” … You [could] tell they [were] totally… like a deer in headlights… [If] people had just a base level of being able to respond to these things really well and it was like a uniform thing across all people who are in a supervisory role, I think that'd be really, really good.’ (I3).

The above quote represents the failure of adequate training for medical supervisors. The scenario described by I3 must have been associated with difficulty and may have discouraged them from mentioning any mental health difficulties again.

## Meta-Theme 7: Preventative factors

### Preventative factors as a barrier

Some interviewees revealed that they would prefer to independently manage their issues rather than seeking help and would only seek help if the issue was severe; this could be a risk factor for mental health issues.

‘My personality is not very conducive. If I’m honest, I tend to shut down [rather than seeking help]. If I’m stressed in any way. I’m very able to compartmentalise. So, I would just block that out. And it’s I know it’s a flaw of my character, because I’m not sure it’s always a very good thing.’ (I28).

This doctor describes how they would be more comfortable’blocking out’ negative thoughts rather than talking about them, which could lead to issues later on.

### Preventative factors as a facilitator

However, this was not necessarily always noted as being a risk factor, in some instances it was protective for doctors because they had positive coping mechanisms which prevented the need to seek help.

‘And meditation and mindfulness just helps me to accept that and concentrate on life in general… But I think that’s what I need to manage my stresses.’ (I20).

Another key protective factor identified was the access to support from informal sources which prevented the need to seek help. This usually came in the form of support from friends and family, who were seen as easier to turn to than colleagues or professional support in times of need.

‘Rather than dealing with things in the workplace, my first port of call is my immediate family. And so always, I think if someone is lucky to have support figures within their family, then that's where someone would naturally often start, rather than escalating immediately to their supervisor.’ (I19).

‘I’m quite well supported; I feel like outside of work. Like not really in professional circles… very much integrated into my community. I go to church. So, I've got a strong group of friends around me and the community around me… I probably have quite a lot of protective factors which means I’m less isolated.’ (I7).

This is not a traditional barrier as shown in the other meta-themes but a protective factor whereby informal support meant that people did not reach the threshold when professional help is required.

### Summary of qualitative study

**Fig. 12:**
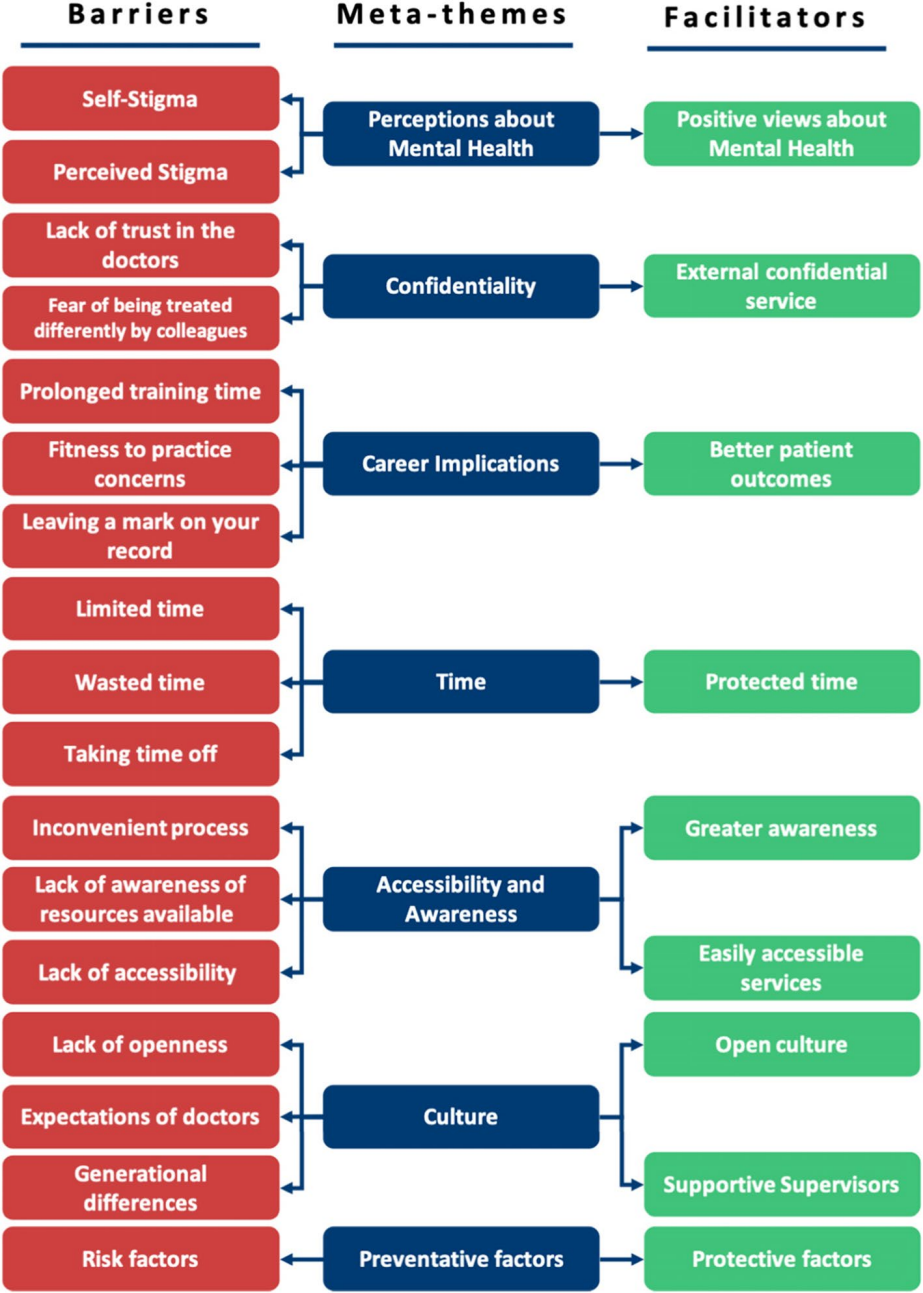
Summary of the meta-themes identified in literature review

In conclusion, our qualitative study fulfilled our objective of identifying the full scope of barriers and facilitators to mental health HSB encountered by NHS doctors. We identified nine novel barriers and facilitators and grouped them into 7 themes. This built upon the existing barriers in the SLR (see Fig. 12) and identified new facilitators that existing literature did not show.

## Discussion

In the SLR, we identified two broad facilitators for mental health HSB: workplace culture and support services. We also found that workplace culture is not always a facilitator and can often cause stigmatisation in seeking care for mental illnesses in medicine, as well as other sectors such as community pharmacy and the military [66, 67, 69]. For this reason, we identified the specific facilitator of *open* culture, and the opposing barrier was *lack* of openness when speaking about mental health.

Under “workplace culture”, we also identified a new facilitator: supportive seniors, which was also recognised as a facilitator to seeking help in active-duty army personnel [69, 70]. Within “perceptions about mental health”, the facilitator found was “positive views about mental health”. Carlton and Deane [71] found that positive attitudes towards help-seeking increased intentions to seek help among those who were at risk of suicide. This contrasted with the barrier of negative views about mental health; where interviewees stated that they may view themselves as weak for seeking help.

Given the stigma around mental health, confidential and external services were mentioned numerous times as a way that would facilitate doctors in seeking help.. This was shown to be a common theme amongst senior doctors in the literature review [72].

The facilitator “improved patient outcomes” was also identified, where interviewees felt seeking help themselves would help them to take better care of their patients. There is research to support the view that doctors caring for themselves can be linked to caring for their patients better [73, 74].

With the meta-theme “time”, the idea of protected time was suggested to potentially aid in help-seeking for doctors. Much like time is booked out of junior doctors' work schedules for teaching and education, time for well-being could be scheduled,such as Balint groups, Schwartz rounds and debrief sessions.

A lack of accessibility and awareness was another significant barrier. Within this theme, we identified two facilitators: greater awareness and easily accessible services. Previously, increased awareness of counselling services has been linked to greater use of those services [75].

### Implications for future research

Understanding the factors that contribute to attitudes to seeking help amongst different medical and surgical specialities.

Attitudes towards seeking help for mental health may vary between speciality, as different specialities may attract doctors of different personality traits [76–78]. Research into speciality-specific barriers could lead to greater personalisation of implementations for different specialities.

- Investigating attitudes to seeking help amongst different HCPs

Future studies could explore the impact of mental ill- health on HCPs, such as nurses and their HSB. The role of personality on HSB in these HCPs could also be explored, leading to implementations to encourage positive attitudes towards help-seeking in HCPs.

- Help-seeking in medical students and mental health teaching in the medical curriculum

There are high rates of mental health issues in medical students, and the BMA have called for better mental health support for medical students [79, 80]. Therefore, exploring the barriers that medical students face to seeking help could lead to greater understanding of how to overcome these barriers and help to shape medical school curricula.

### Limitations

#### Systematic literature review limitations

Although great effort was taken to ensure the inclusion and exclusion criteria were comprehensive, they may have restricted the number of articles that were considered and cause exclusion of relevant papers [81]. Despite HCPs being the focus of the SLR, most of the articles discussed the barriers and facilitators faced by doctors and these may not be generalisable to those faced by all HCPs. In all articles, data collection relied on self-reported measures, which may have led to social desirability and recall bias.

#### Primary data collection limitations

Firstly, the sample size was not large enough to measure the impact of confounding variables such as age, and social desirability bias, as well as self-selection bias may have occurred due to the self-reporting nature of the data collection.

Due to ethical limitations, we could not ask doctors about previous mental health diagnoses. Future research could focus on doctors that have sought help and explore the key factors that encouraged them to do so. Due to the time and resource constraints of the project, as well as the COVID-19 pandemic increasing the working hours of our target population [82, 83], the number of doctors we could recruit was limited.

Future studies should aim to take place over a longer length of time and target a larger sample size and reduce voluntary recruitment to try to decrease non-responder bias [84].

## Conclusion

Our project aimed to investigate the barriers and facilitators perceived by NHSdoctors when seeking mental health support. Our SLR identified nine barriers and two facilitators. Our primary data collection then elicited novel barriers and facilitators.

With the COVID-19 pandemic shining a light on doctors and increasing awareness of their difficult working lives, we hope that the culture in the NHS will continue to change, to view seeking help positively and to aid doctors in overcoming the barriers they face. We hope that our research will allow doctors to be celebrated in the way they truly deserve, by recognising their role as healers, who need to first heal themselves, before they are able to heal others.  

## Supplementary Information

**Additional file 1:** **Table S1.** Search string in MEDLINE database. **Table S2. **Search string in EMBASE database. **Table S3.** Search string in PsychInfo database. **Table S4.** Search string in HMIC database.

**Additional file 2.** A summary of the studies included in the systematic literature review.

**Additional file 3.** Interview guide.

**Additional file 4.** Critical appraisal checklists.

**Additional file 5.** Interview participant information sheet.

## Data Availability

The datasets used and/or analysed during the current study are available from the corresponding author on reasonable request.

## Abbreviations

MH: Mental Health
HSB: Help-Seeking Behaviour
HCP: Healthcare Professionals
HBM: Health Belief Model
BMA: British Medical Association
BMJ: British Mediccal Journal
CEBMa: Centre for Evidence-Based Management
EMBASE: Exceptra Medica Database
MEDLINE: Medical Literature Analysis and Retrieval System Online
APA PsycINFO: American Psychological Association PsycINFO
GP: Genral Practitioner
HMCI: Healthcare Management Information Consortium
ICREC: Imperial College Research and Ethics Committee
NHS: National Health Service
SLR: Systematic Leterature Review
PICO: Patient, Intervention, Comaprison and Outcome
PRISMA: Preferred Reporting Items for Systematic Reviews an Meta-Analysis
SSI: Semi Structured Interviews
GMC: General Medical Council
