# Supplementary material for: What are the barriers and facilitators to seeking help for mental health in NHS doctors: a systematic review and qualitative study
Source: BMC Psychiatry. 2022 Sep 7;22:595. doi: 10.1186/s12888-022-04202-9 (PMC9450826; doi:10.1186/s12888-022-04202-9)
Supplement: Supplementary file 1 — Additional file 1: Table S1. Search string in MEDLINE database. Table S2. Search string in EMBASE database. Table S3. Search string in PsychInfo database. Table S4. Search string in HMIC database. [file 12888_2022_4202_MOESM1_ESM.docx]

Table S1 - Search string in MEDLINE database

| Search # | Searches | Results | Database |
| --- | --- | --- | --- |
| 1 | exp Mental Health/ | 41633 | MEDLINE |
| 2 | mental wellbeing.mp. | 889 | MEDLINE |
| 3 | mental well-being.mp. | 2782 | MEDLINE |
| 4 | Exp Mental Disorders/ | 1269353 | MEDLINE |
| 5 | 1 or 2 or 3 or 4 | 1299157 | MEDLINE |
| 6 | (help seek$ or seek$ help or seek$ treatment).mp. [mp=title, abstract, original title, name of substance word, subject heading word, floating sub-heading word, keyword heading word, organism supplementary concept word, protocol supplementary concept word, rare disease supplementary concept word, unique identifier, synonyms] | 15949 | MEDLINE |
| 7 | Exp Help-Seeking Behavior/ | 867 | MEDLINE |
| 8 | (doctors or nurses or physicians or healthcare workers or healthcare practitioners or healthcare professionals or physiotherapists or allied healthcare or healthcare staff).mp. [mp=title, abstract, original title, name of substance word, subject heading word, floating sub-heading word, keyword heading word, organism supplementary concept word, protocol supplementary concept word, rare disease supplementary concept word, unique identifier, synonyms] | 719600 | MEDLINE |
| 9 | 6 or 7 | 15949 | MEDLINE |
| 10 | 5 and 8 and 9 | 498 | MEDLINE |
| 11 | Limit 10 to english language | 475 | MEDLINE |

***Final article number: 475***

Table S2 - Search string in EMBASE database

| Search # | Searches | Results | Database |
| --- | --- | --- | --- |
| 1 | exp Mental Health/ | 175693 | EMBASE |
| 2 | Exp psychological well-being/ | 20822 | EMBASE |
| 3 | Exp mental disease/ | 2432502 | EMBASE |
| 4 | mental wellbeing.mp. | 1352 | EMBASE |
| 5 | mental well-being.mp. | 3453 | EMBASE |
| 6 | 1 or 2 or 3 or 4 or 5 | 2520054 | EMBASE |
| 7 | (help seek$ or seek$ help or seek$ treatment).mp. [mp=title, abstract, heading word, drug trade name, original title, device manufacturer, drug manufacturer, device trade name, keyword, floating subheading word, candidate term word] | 29019 | EMBASE |
| 8 | exp help seeking behavior/ | 12325 | EMBASE |
| 9 | (doctors or nurses or physicians or healthcare workers or healthcare practitioners or healthcare professionals or physiotherapists or allied healthcare or healthcare staff).mp. [mp=title, abstract, heading word, drug trade name, original title, device manufacturer, drug manufacturer, device trade name, keyword, floating subheading word, candidate term word] | 747844 | EMBASE |
| 10 | help seeking behaviour.mp. | 927 | EMBASE |
| 11 | 7 or 8 or 10 | 29019 | EMBASE |
| 12 | 6 and 9 and 11 | 1012 | EMBASE |
| 13 | Limit 12 to english language | 961 | EMBASE |

***Final article number: 961***

Table S3 - Search string in PsychInfo database

| Search # | Searches | Results | Database |
| --- | --- | --- | --- |
| 1 | exp Mental Health/ | 69975 | PyschInfo |
| 2 | mental wellbeing.mp. | 576 | PyschInfo |
| 3 | mental well-being.mp. | 2525 | PyschInfo |
| 4 | Exp Mental Disorders/ | 878932 | PyschInfo |
| 5 | 1 or 2 or 3 or 4 | 929574 | PyschInfo |
| 6 | (help seek$ or seek$ help or seek$ treatment).mp. [mp=title, abstract, heading word, table of contents, key concepts, original title, tests & measures, mesh] | 18499 | PyschInfo |
| 7 | exp Help Seeking Behavior/ or exp Health Care Seeking Behavior/ | 14044 | PyschInfo |
| 8 | (doctors or nurses or physicians or healthcare workers or healthcare practitioners or healthcare professionals or physiotherapists or allied healthcare or healthcare staff).mp. [mp=title, abstract, heading word, table of contents, key concepts, original title, tests & measures, mesh] | 125704 | PyschInfo |
| 9 | 6 or 7 | 25348 | PyschInfo |
| 10 | 5 and 8 and 9 | 592 | PyschInfo |
| 11 | Limit 10 to english language | 563 | PyschInfo |

***Final article number: 563***

Table S4 - Search string in HMIC database

| Search # | Searches | Results | Database |
| --- | --- | --- | --- |
| 1 | exp Mental Health/ | 6528 | HMIC |
| 2 | mental wellbeing.mp. | 165 | HMIC |
| 3 | mental well-being.mp. | 154 | HMIC |
| 4 | Exp Mental illlness/ | 7138 | HMIC |
| 5 | 1 or 2 or 3 or 4 | 12851 | HMIC |
| 6 | (help seek$ or seek$ help or seek$ treatment).mp. [mp=title, other title, abstract, heading words] | 693 | HMIC |
| 7 | exp Access to health services/ | 6902 | HMIC |
| 8 | help seeking.mp. | 305 | HMIC |
| 9 | exp Health Behaviour/ | 1624 | HMIC |
| 10 | 6 or 7 or 8 or 9 | 9034 | HMIC |
| 11 | (doctors or nurses or physicians or healthcare workers or healthcare practitioners or healthcare professionals or physiotherapists or allied healthcare or healthcare staff).mp. [mp=title, other title, abstract, heading words] | 45028 | HMIC |
| 12 | 5 and 10 and 11 | 43 | HMIC |

***Final article number: 43***

*The search string above details the strategy used to discover the relevant articles for the Systematic Literature Review. The databases searched include MEDLINE, EMBASE, PyschInfo and HMIC.*
