# Supplementary material for: What are the barriers and facilitators to seeking help for mental health in NHS doctors: a systematic review and qualitative study
Source: BMC Psychiatry. 2022 Sep 7;22:595. doi: 10.1186/s12888-022-04202-9 (PMC9450826; doi:10.1186/s12888-022-04202-9)
Supplement: Supplementary file 2 — Additional file 2. A summary of the studies included in the systematic literature review. [file 12888_2022_4202_MOESM2_ESM.docx]

| **First Author, Year, Country** | **Study Design** | **Sample Characteristics and Size** | **Barriers** | **Facilitators** | **Critical Appraisal** |
| --- | --- | --- | --- | --- | --- |
| (Adams *et al.*, 2010) (34)  UK | Cross- sectional survey | GPs (n=1122) and psychiatrists (n=132) | Stigma, negative career implications, confidentiality concerns, letting colleagues down (time related presenteeism), letting patients down (time related presenteeism). | N/A | Study is localised to two areas so may not be generalisable. Self-reporting may have caused social desirability or recall bias. |

*A summary of the studies included in the systematic literature review.*

| **First Author, Year, Country** | **Study Design** | **Sample Characteristics and Size** | **Barriers** | **Facilitators** | **Critical Appraisal** |
| --- | --- | --- | --- | --- | --- |
| (Axisa *et al.*, 2019) (35) Australia | Cross- sectional survey | Trainee Doctors (n=59) | Negative career implications, confidentiality concerns, embarrassment (self-stigma), sign of weakness, time constraints. | N/A | Small sample size limits the generalisability of the findings. The lack of analysis on non- responders causes non- response bias. |
| (Baldwin, Dodd & Wrate, 1997) (48)  UK | Cohort study survey | Junior doctors (n=142) | Self-treatment (preventing actions), lack of awareness of services. | N/A | Sample is localised to one area and may not be generalisable. Self-reporting may have caused social desirability or recall bias. |
| (Bianchi, Bhattacharyya & Meakin, 2016) (51)  UK | Cross- sectional interviews | Doctors (n=14) | Stigma, negative career implications, vulnerability (self- stigma), fear of prejudice (perceived stigma), denial/lack of illness recognition. | Organisational and team support, awareness of support services. | Results may not be representative of the population due to the small sample size.  Participants were recruited from one location so views may not be generalisable. Participants partaking due to personal reasons may have resulted in self-selection bias. |

| **First Author, Year, Country** | **Study Design** | **Sample Characteristics and Size** | **Barriers** | **Facilitators** | **Critical Appraisal** |
| --- | --- | --- | --- | --- | --- |
| (Edwards & Crisp, 2017) (38)  Australia | Cross- sectional survey | MH professionals and clinicians in training (n=98) | Negative career implications, confidentiality concerns, embarrassment, sign of weakness, concerns for colleague's views and time constraint, self-treatment, financial reasons. | N/A | The use of snowball sampling method and self-selection may have over-represented the findings. Due to the small sample size, findings may not be a true representation of the population of MH professionals. |
| (Gold *et al.*, 2016) (45) USA | Cross- sectional survey | Female doctors (n=2106) | Stigma, negative career implications, time constraint, embarrassment, lack of illness recognition, belief that they could get through this without help, lack of awareness of support services, financial reasons. | N/A | Sample obtained from social media so the group who responded may be younger than the average female doctor in the USA. Responders and non-responders cannot be compared to check for non- response bias. The sample only focuses on mothers and therefore this can have a confounding effect on the results. |

| **First Author, Year, Country** | **Study Design** | **Sample Characteristics and Size** | **Barriers** | **Facilitators** | **Critical Appraisal** |
| --- | --- | --- | --- | --- | --- |
| (Jones, Whybrow & Coetzee, 2018) (53)  UK | Comparative cohort study survey | Military doctors (n=678) and ex- military personnel (n=1448) | Stigma, self-stigma, time constraints, self-management. | N/A | Differing sample size and response rates between comparative groups may have introduced bias. Uncollectable data from non-respondents may have caused non-response bias. |
| (Lee, Jeong & Yi, 2020) (52)  South Korea | Cross- sectional survey | Nurses (n=184) | Self-stigma, public stigma, inertia resulting from depression. | N/A | The sample met the required size for a significant result to be generated. Self-reporting may have caused social desirability or recall bias. |
| (Rees *et al.*, 2019) (37) UK | Cross- sectional interview | Doctors (n=42) and medical students (n=4) | Stigma, negative career implications, confidentiality concerns, seen as a weakness, lack of illness recognition, difficulty to disclose when suffering. | N/A | Purposive sampling may have caused self-selection-bias and introduced themes inconsistent with the population. |

| (Schwenk, Gorenflo & Leja, 2008) (36)  Schwenk | Cross- sectional survey | Doctors (n=1154) | Stigma, confidentiality concerns, negative career implications, self-treatment (preventing actions). | N/A | The low response rate and self- reporting may mean the data is not generalisable. Uncollectable data from non-respondents may have caused non-response bias. |
| --- | --- | --- | --- | --- | --- |

| **First Author, Year, Country** | **Study Design** | **Sample Characteristics and Size** | **Barriers** | **Facilitators** | **Critical Appraisal** |
| --- | --- | --- | --- | --- | --- |
| (Spiers *et al.*, 2017) (42) United Kingdom | Cross- sectional interviews | GPs (n=47) | Stigma, privacy and confidentiality concerns, time constraints, guilt-induced presenteeism (time), lack of access to adequate treatment (accessibility), inability to move from doctor to patient role (negative evaluation of therapy), feelings of inertia and denial (preventing actions). | Openness between colleagues, emotional support from colleagues and availability of a specialist, confidential service. | The self-selection sampling may translate to over- representation. |
| (Van der Bijl & Oosthuizen, 2007) (57) South Africa | Cross- sectional surveys | Doctors (n=135) | Self-treatment (preventing actions). | N/A | Sample from one localised area may mean results cannot be generalised. Self-selection due to fear of being identified may lead to under-representation. |

| **First Author, Year, Country** | **Study Design** | **Sample Characteristics and Size** | **Barriers** | **Facilitators** | **Critical Appraisal** |
| --- | --- | --- | --- | --- | --- |
| (West *et al.*, 2016) (54) USA | Cross- sectional survey | Doctors (n=5829) | Negative career implications. | N/A | Analysis of response bias showed the sample was representative with respect to demographics but, lack of information on HSB of non- responders limits the representativeness of MH HSB. |
| (White *et al.*, 2018) (33) UK | Cross- sectional survey | Psychiatrists (n=370) | Stigma, negative career implications, confidentially concerns, professional integrity (confidentiality). | Confidential services. | The study is localised to one area and therefore may not be generalisable. Self-selection may have caused over- representation. |
| (Worley, 2008) (55) USA | Cross- sectional viewpoints | Medical students and doctors (n=8) | Negative career implications. | N/A | Small sample size and the viewpoints from medical students and doctors cannot be distinguished. |

| **First Author, Year, Country** | **Study Design** | **Sample Characteristics and Size** | **Barriers** | **Facilitators** | **Critical Appraisal** |
| --- | --- | --- | --- | --- | --- |
| ((Teo1 *et al.*, 2021)) (44)  Singapore | Cross- sectional survey | Allied HCPs (n=328) | Stigma, time constraints, negative evaluation of therapy, lack of motivation, emotional concerns, time constraints, stigma, misfit of therapy needs (negative evaluation of therapy), availability of services, cost. | N/A | The sample size did not meet the minimum required participants (348) needed to be representative of the population. The low response rate (29.1%) may cause non- response bias. Also, social desirability bias may have influenced participant responses. |
