# Supplementary figures and images for: What are the barriers and facilitators to seeking help for mental health in NHS doctors: a systematic review and qualitative study
Source: BMC Psychiatry. 2022 Sep 7;22:595. doi: 10.1186/s12888-022-04202-9 (PMC9450826; doi:10.1186/s12888-022-04202-9)

**Interview guide**

***The interview questions followed the structure below:***


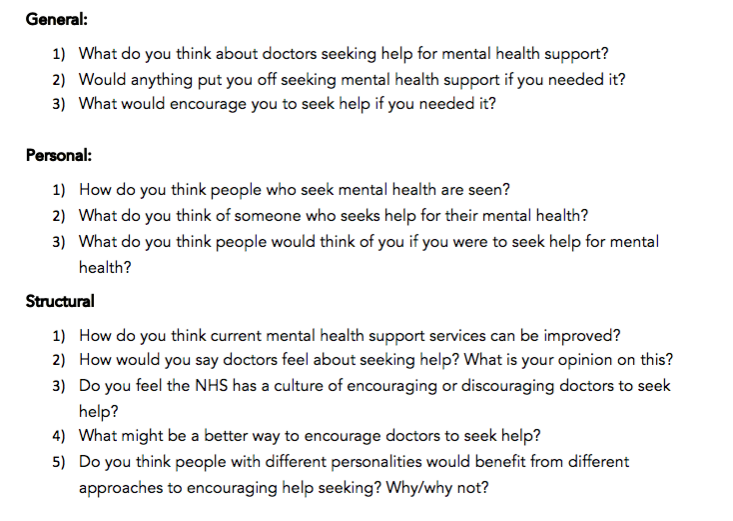

Supplement: Supplementary file 3 — Additional file 3. Interview guide. [file 12888_2022_4202_MOESM3_ESM.docx]

## Additional file 5 Interview Participant Information Sheet


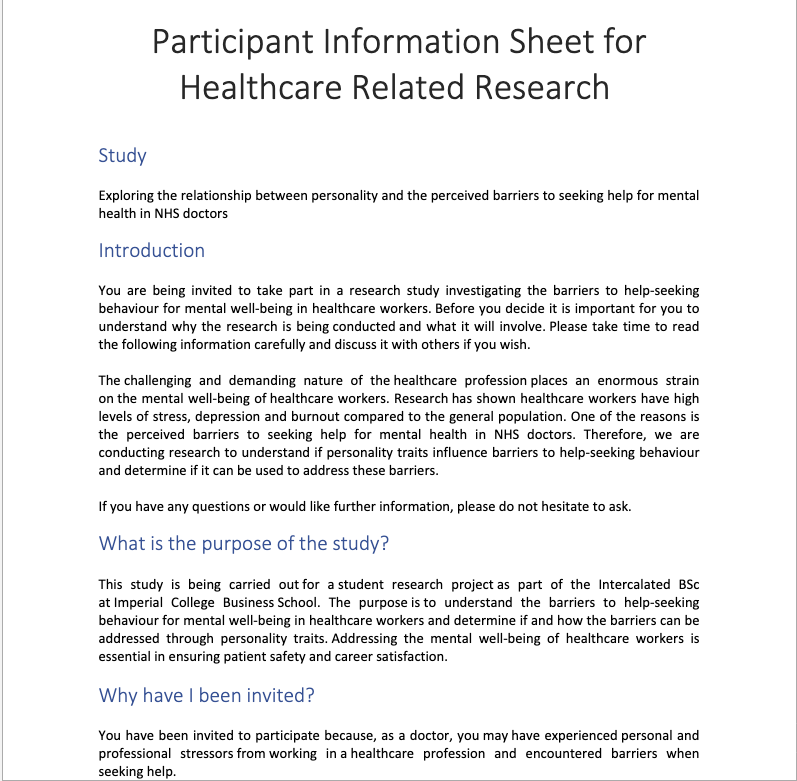


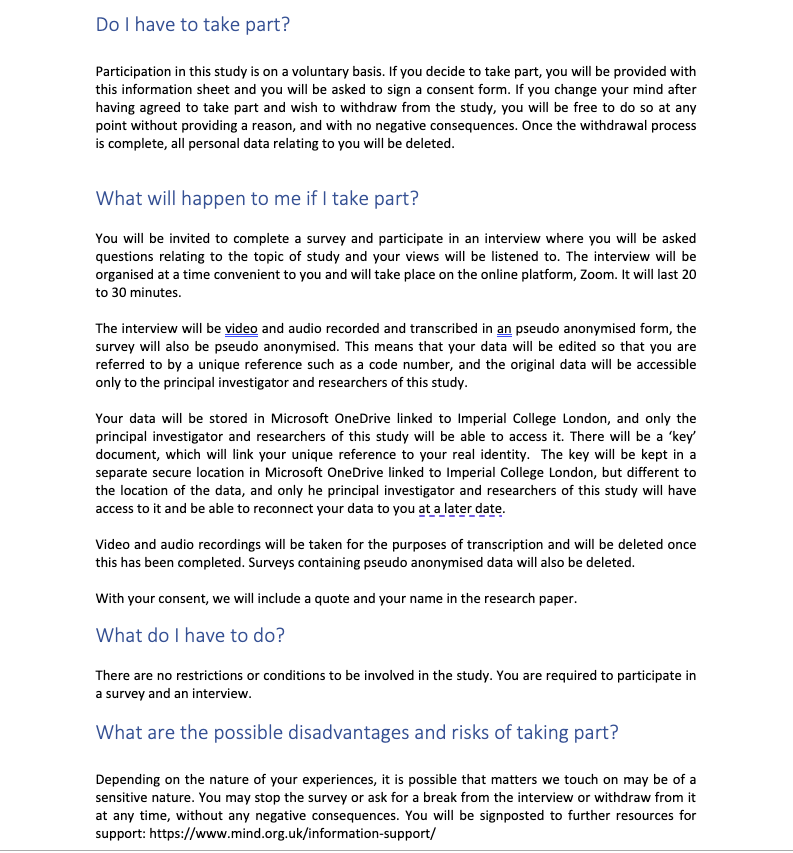


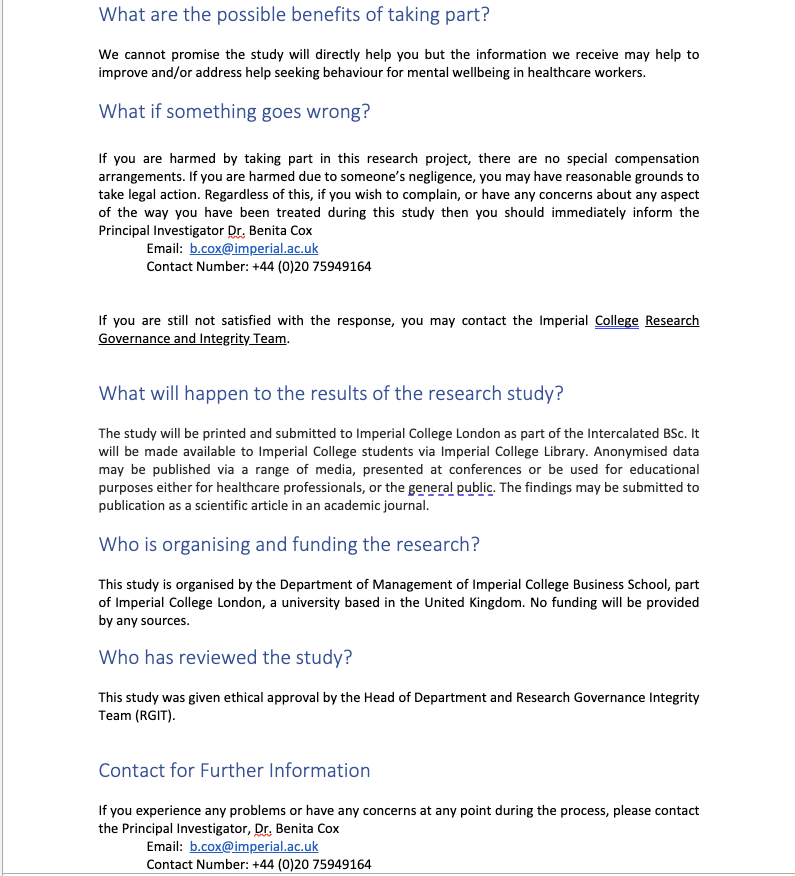


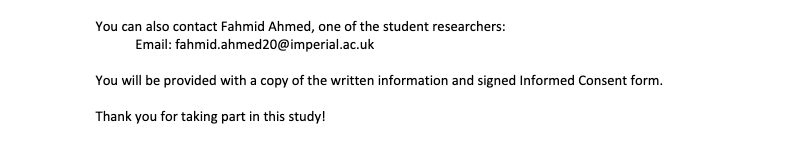


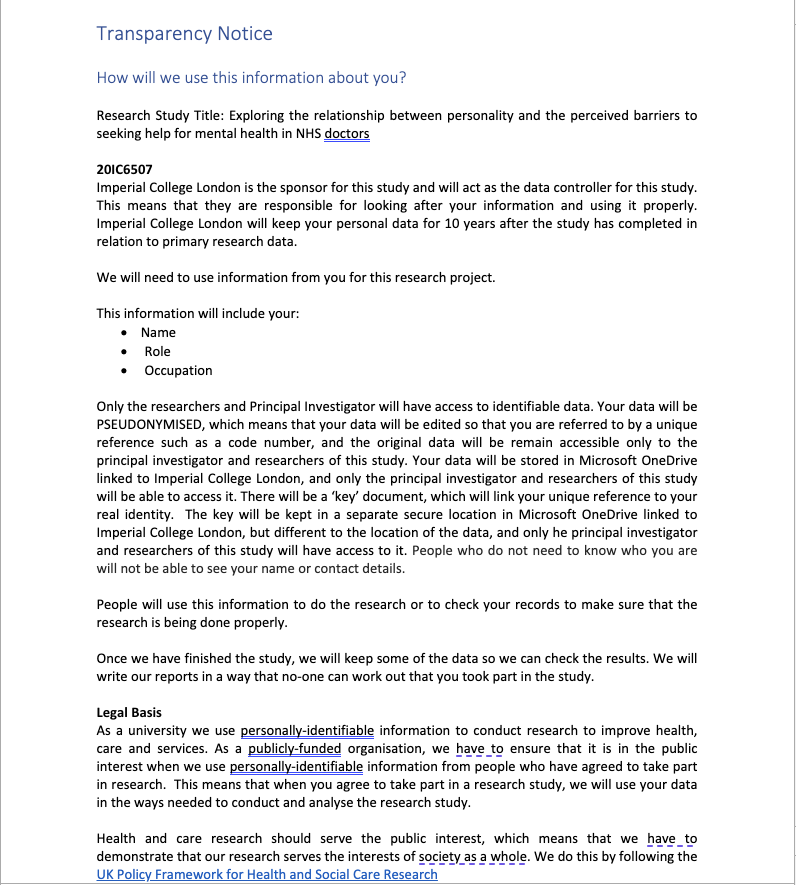


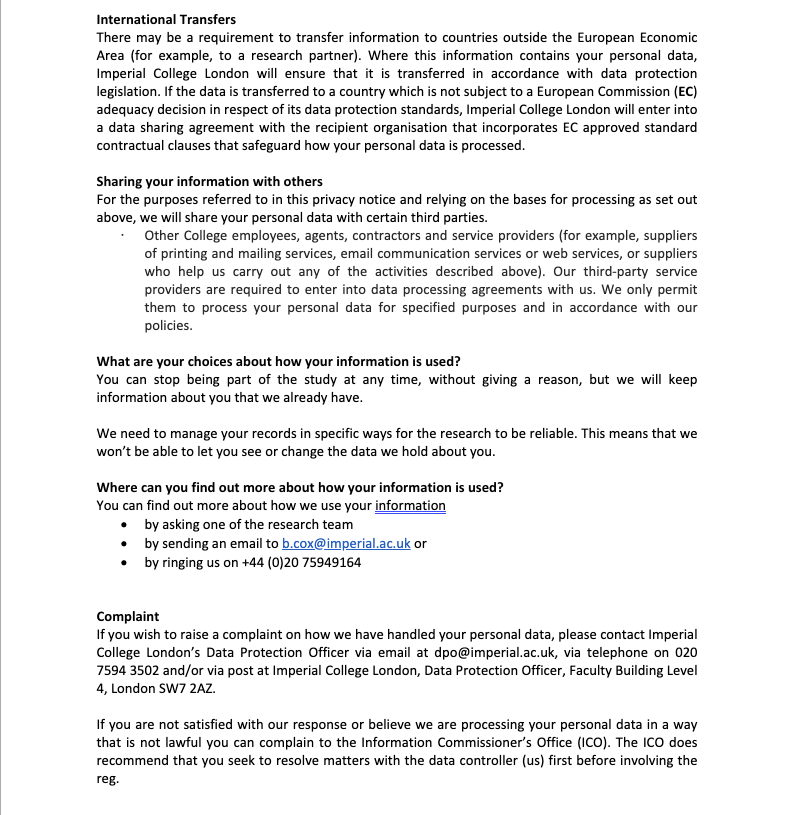

Supplement: Supplementary file 5 — Additional file 5. Interview participant information sheet. [file 12888_2022_4202_MOESM5_ESM.docx]
