# Supplementary material for: What are the barriers and facilitators to seeking help for mental health in NHS doctors: a systematic review and qualitative study
Source: BMC Psychiatry. 2022 Sep 7;22:595. doi: 10.1186/s12888-022-04202-9 (PMC9450826; doi:10.1186/s12888-022-04202-9)

Additional file 4: Critical Appraisal Checklists

Adams et al 2010
What stops us from healing the healers: a Survey of Help-Seeking Behaviour, Stigmatisation and Depression within the medical profession

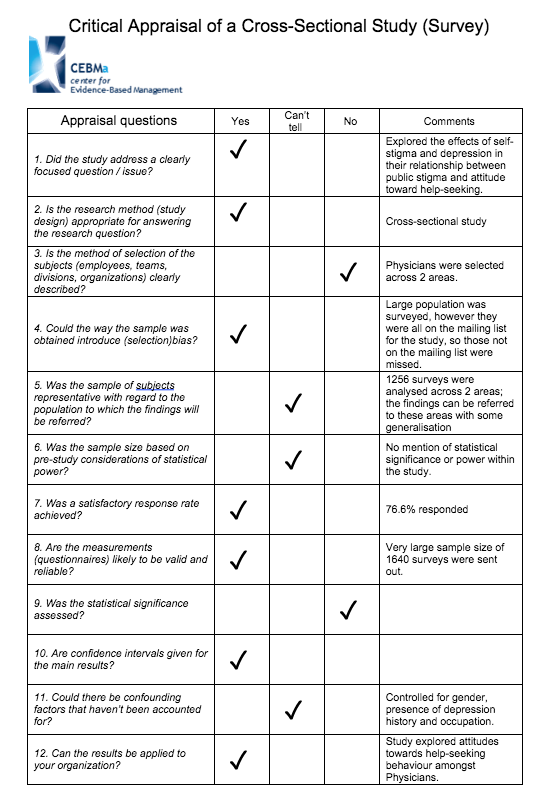


Baldwin et al 1997
Young doctors' health--II. Health and health behaviour

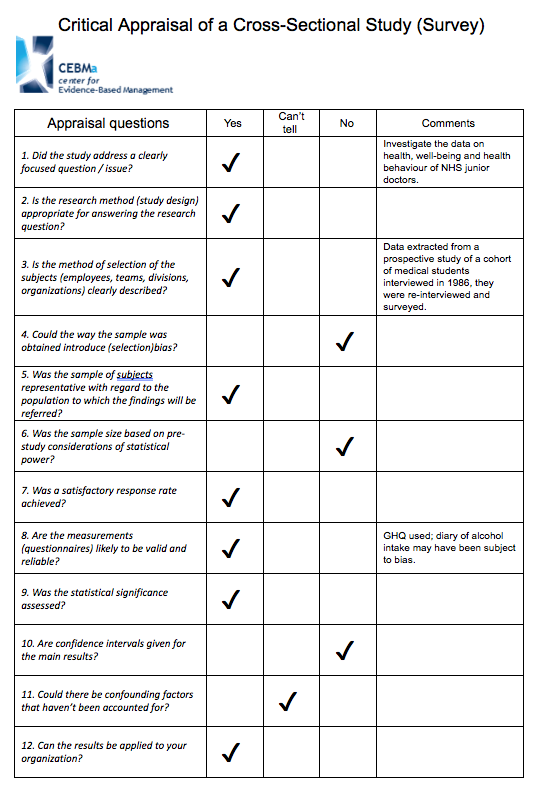


Bianchi et al 2016
Exploring senior doctors’ beliefs and attitudes regarding mental illness within the medical profession: a qualitative study

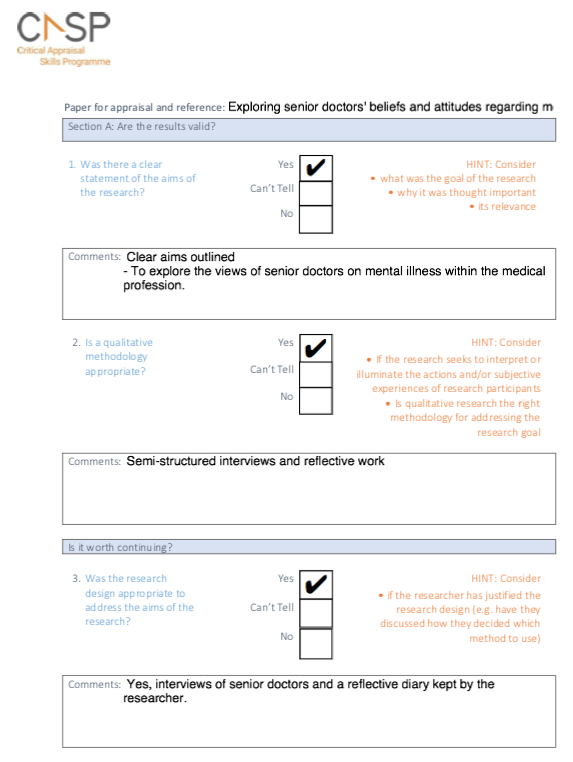

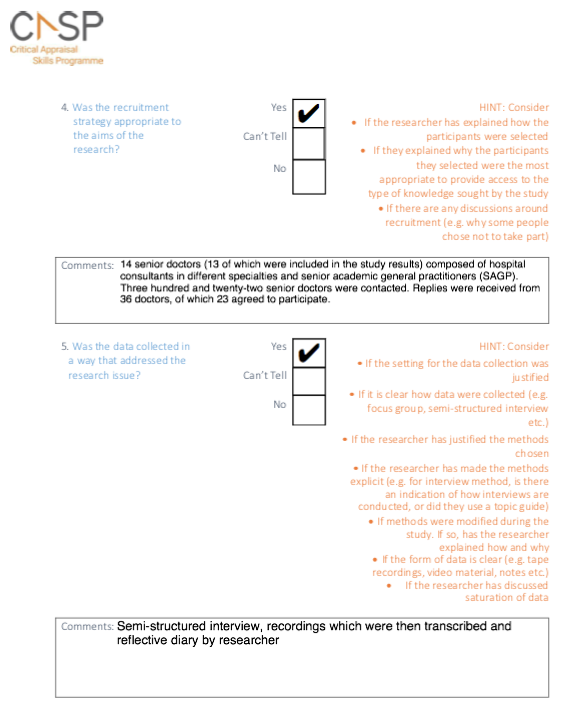

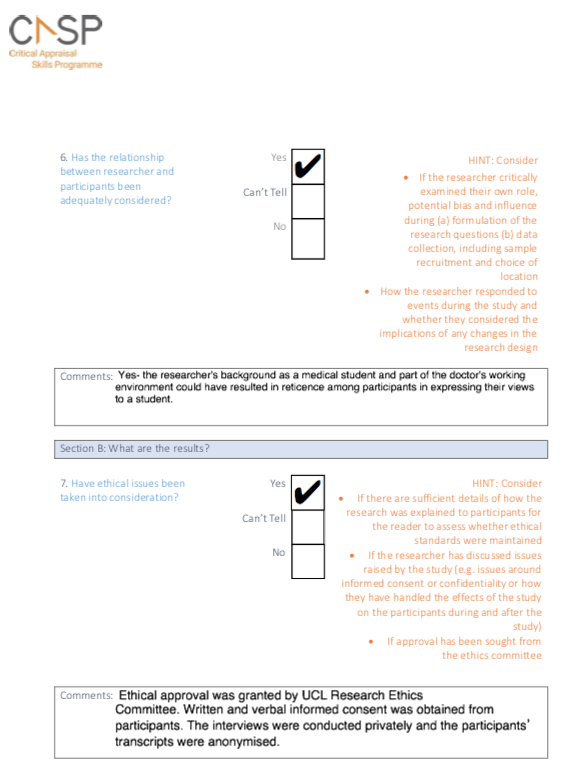

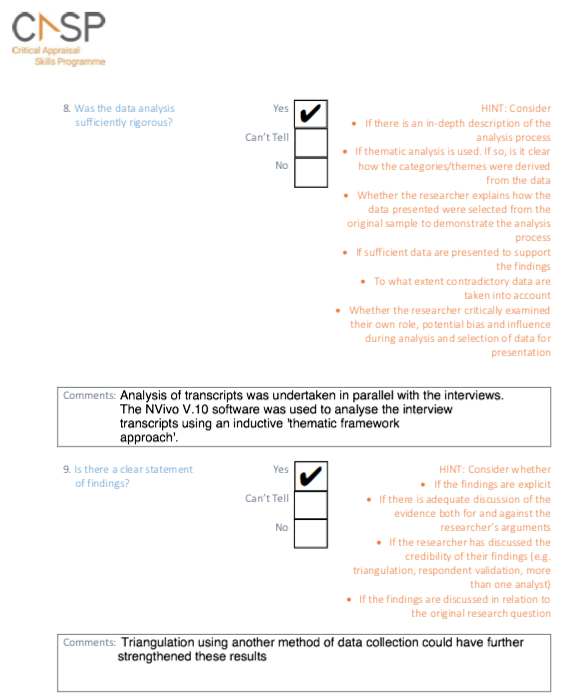

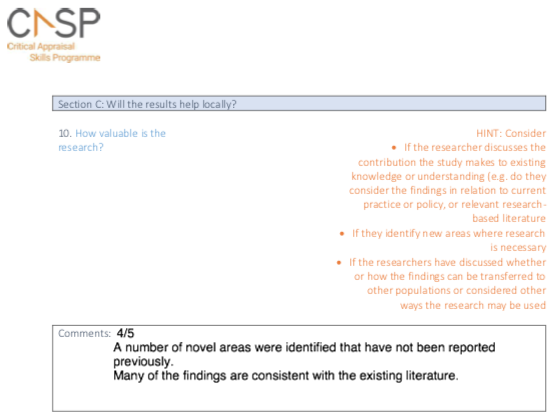


Bijl et al 2007
Anxiety, depression, health attitudes and coping strategies in doctors and teachers in a Cape Town setting


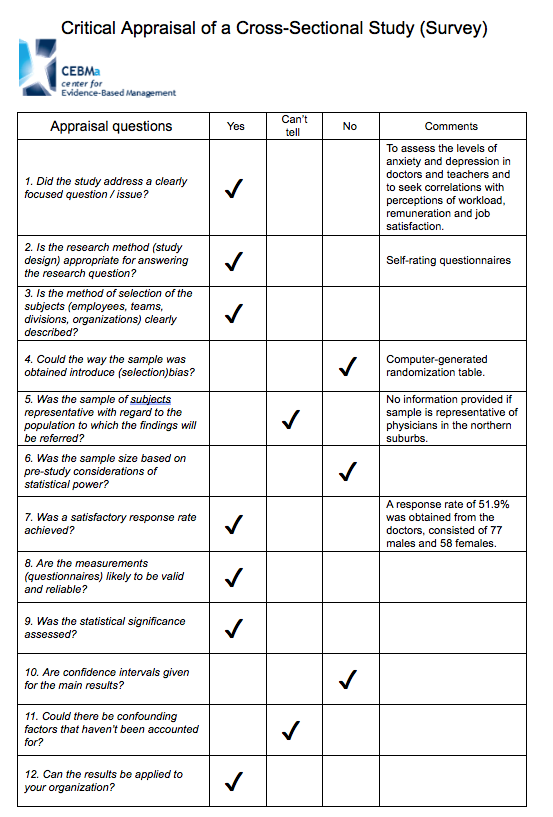


Braquehais et al 2014
Promoting voluntary help-seeking among doctors with mental disorders


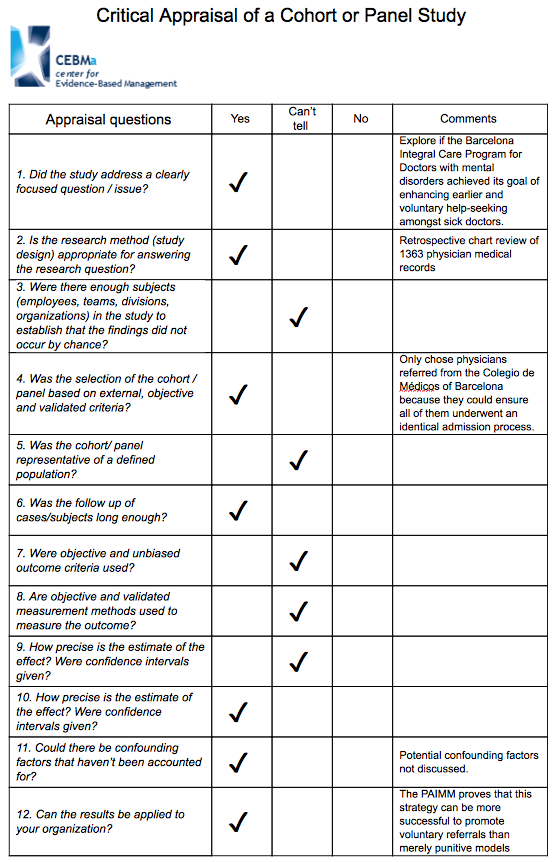


Dyrbye et al 2017
Medical Licensure Questions and Physician Reluctance to Seek Care for Mental Health Conditions


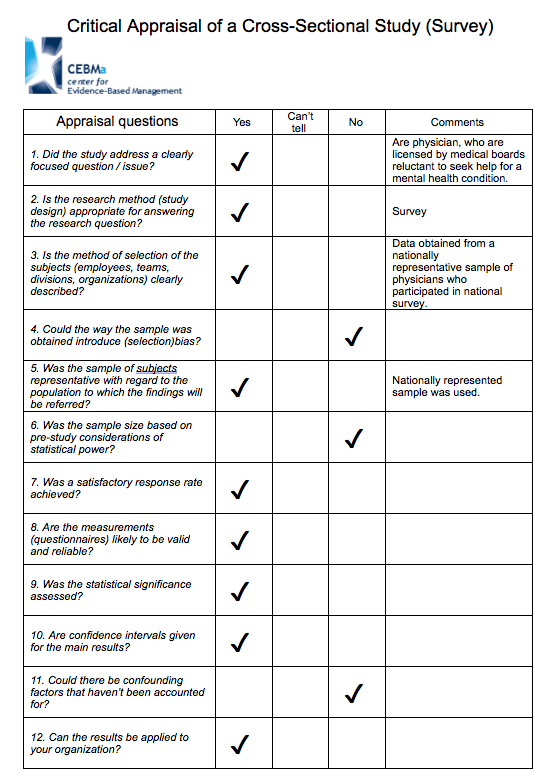


Edwards et al 2016
Seeking help for psychological distress: Barriers for mental health professionals


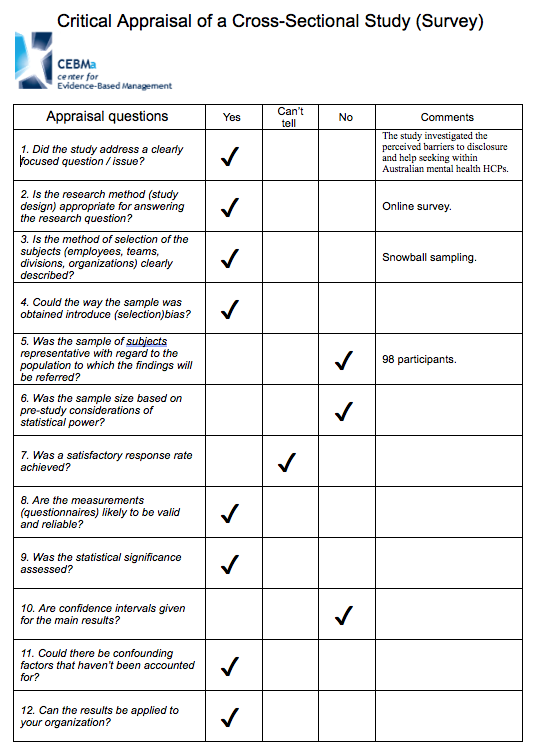


Fridner et al 2012
Why don’t academic physicians seek needed professional help for psychological distress?


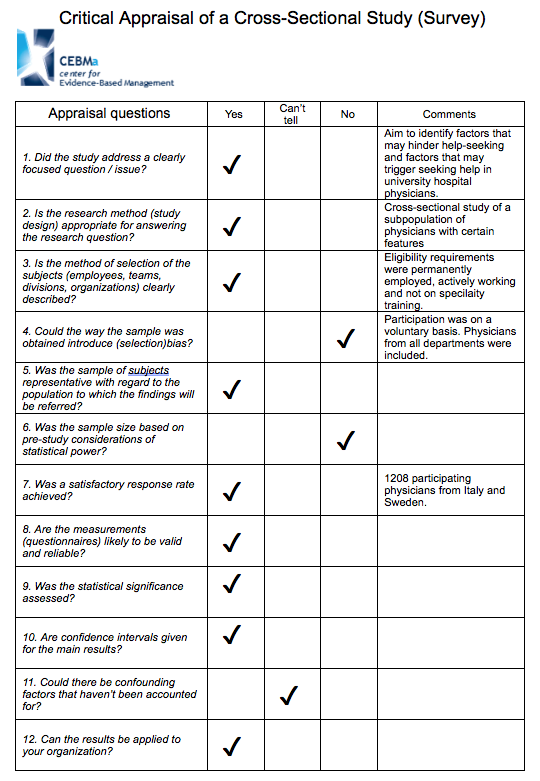


Gold et al 2016
“I would never want to have a mental health diagnosis on my record”: A survey of female physicians on mental health diagnosis, treatment, and reporting


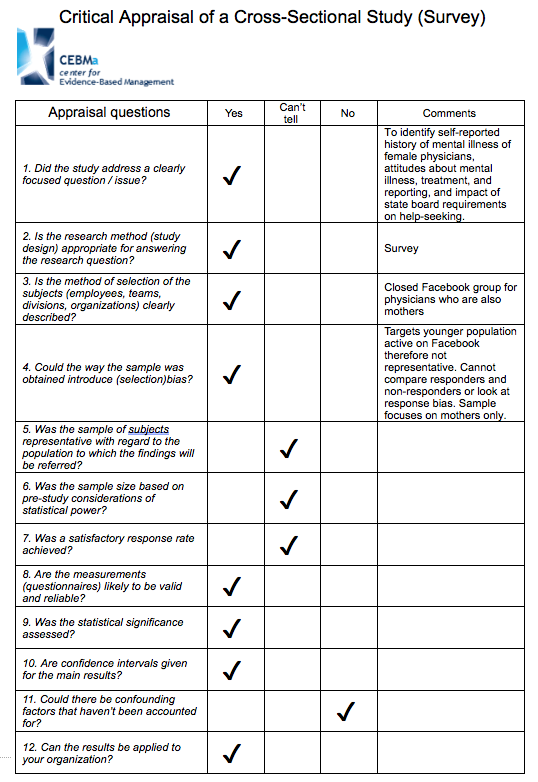


Lee et al 2020
Nurses' attitudes toward psychiatric help for depression: The serial mediation effect of self-stigma and depression on public stigma and attitudes toward psychiatric help


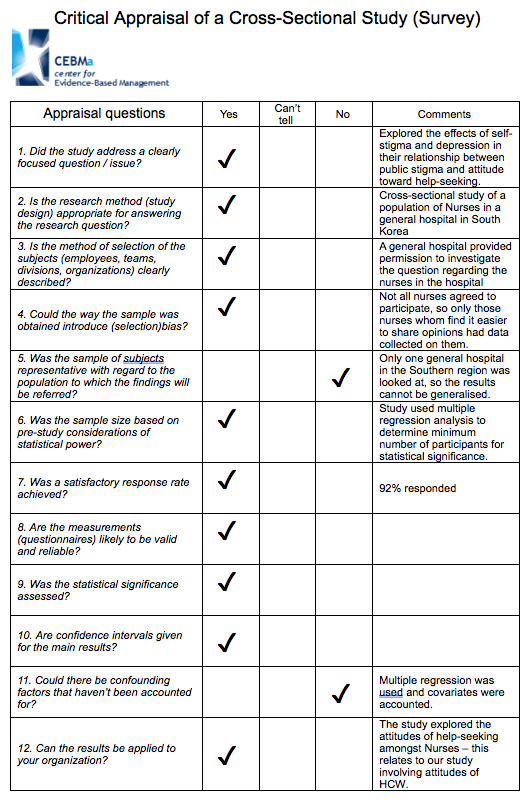


Rennert et al 1990
The Care of Family Physicians and Their Families: A Study of Health and Help-Seeking Behaviour


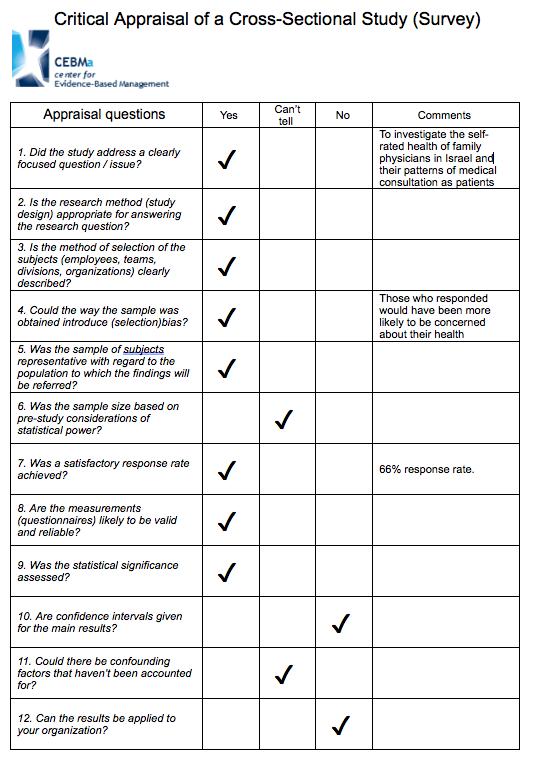


Ress et al 2019
Doctor's decisions when disclosing their mental health


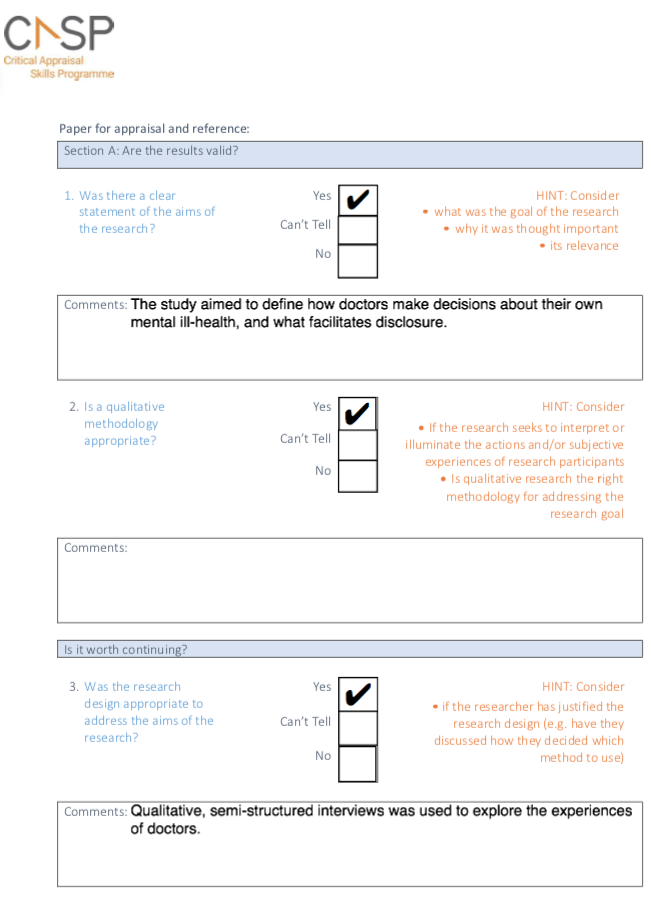


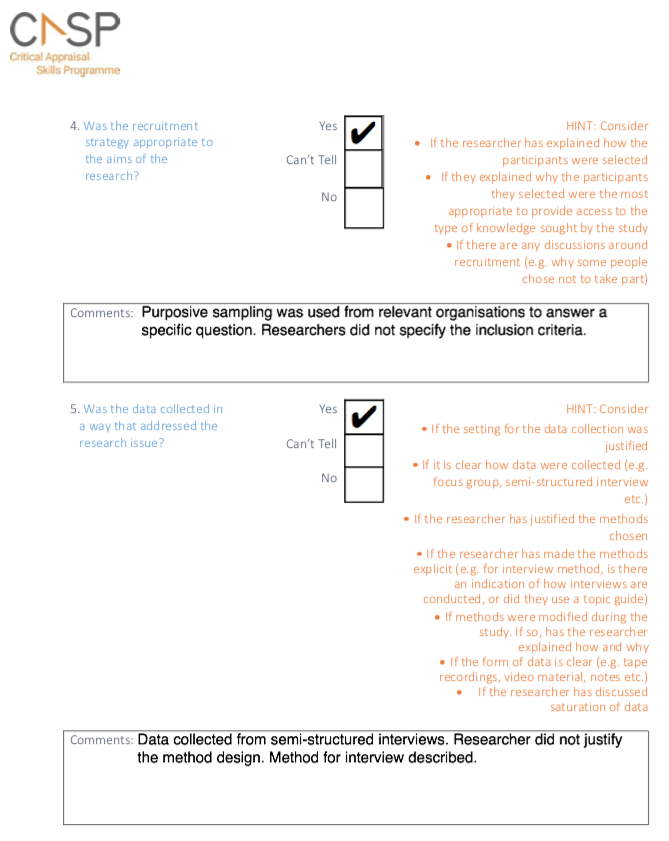

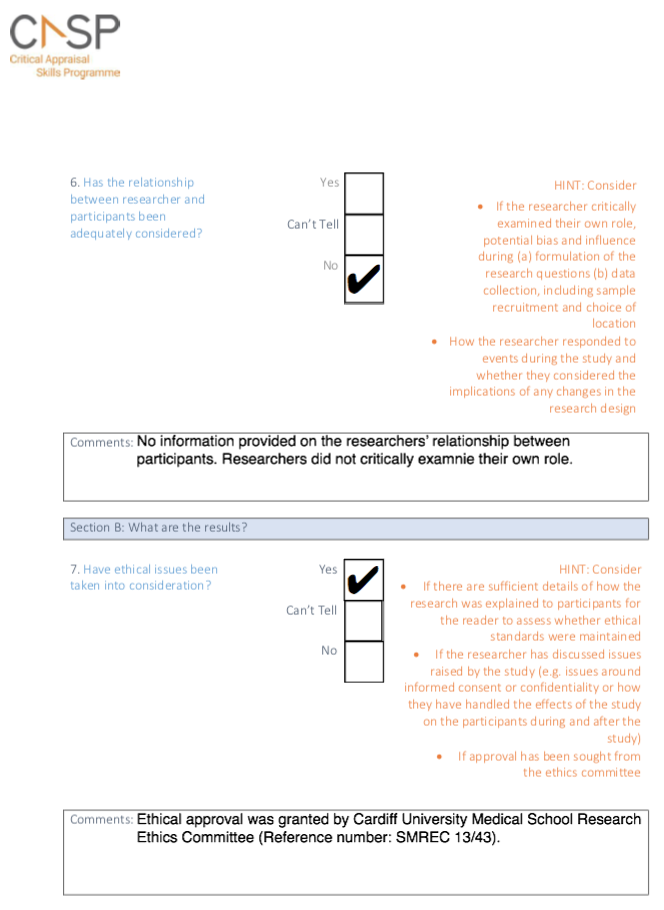

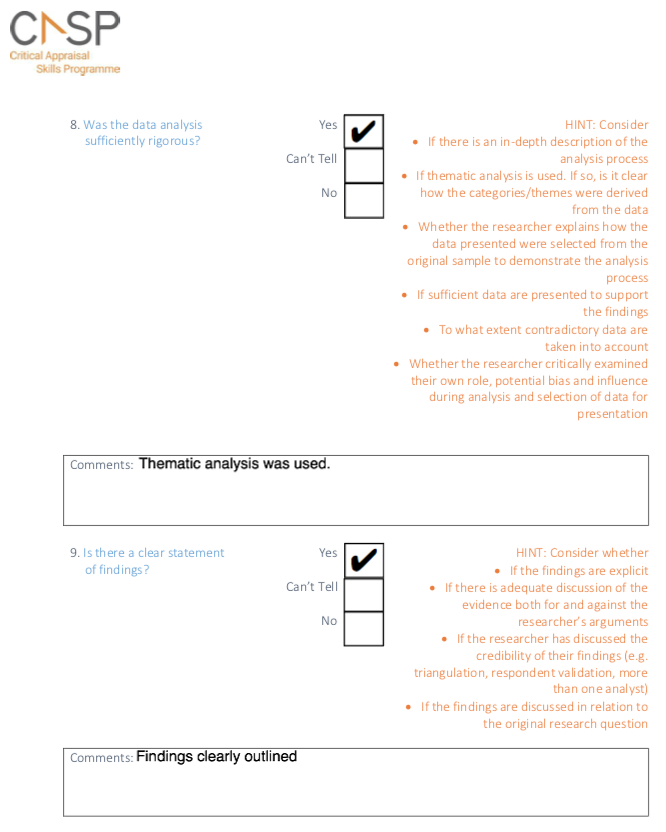

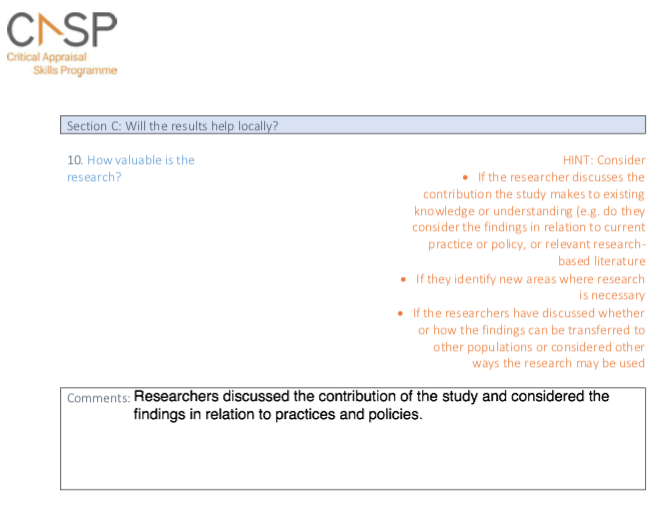


Schneider et al 2007
Personal use of medical care and drugs among Swiss primary care physicians


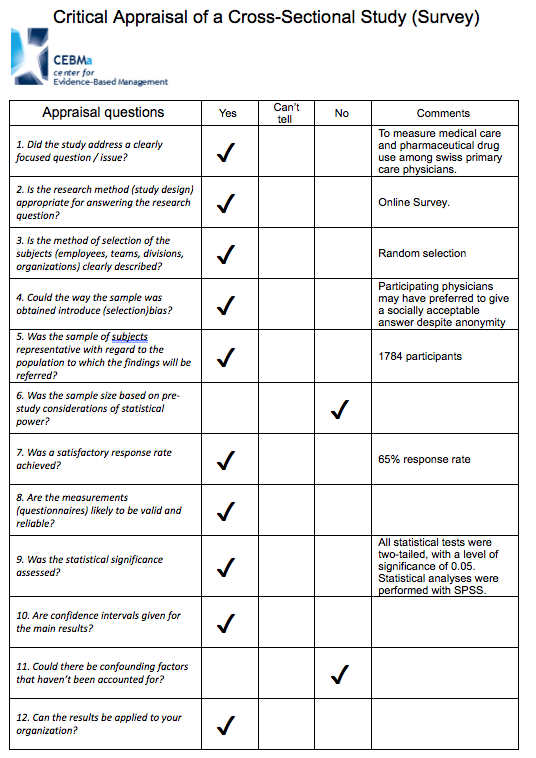


Schwenk et al 2008
A Survey on the Impact of Being Depressed on the Professional Status and Mental Health Care of Physicians


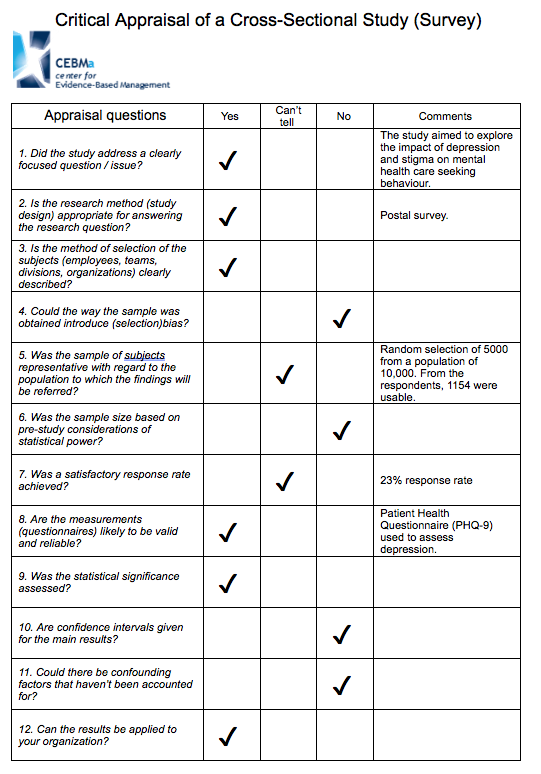


Spiers et al 2017
Barriers, facilitators, and survival strategies for GPs seeking treatment for distress: a qualitative study.


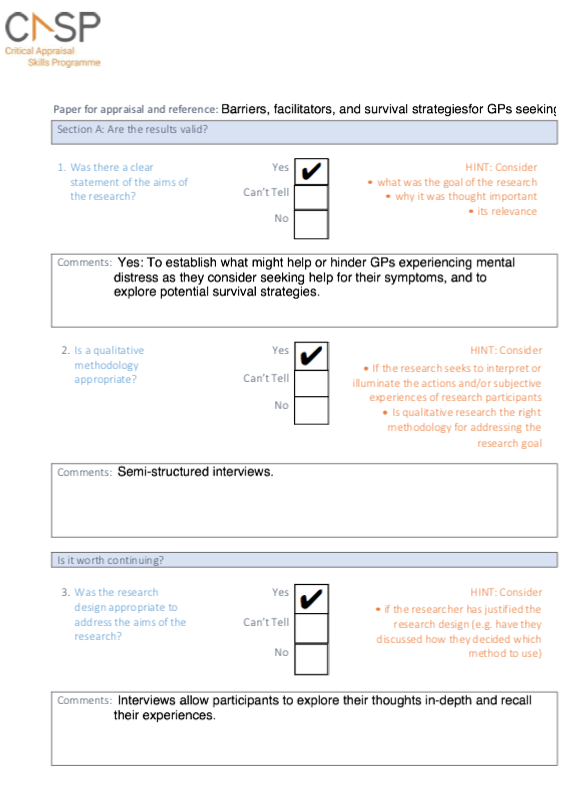


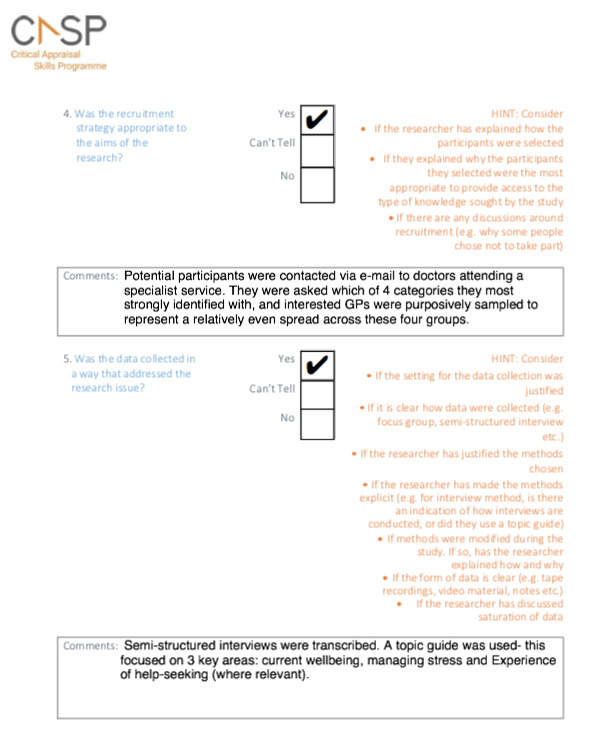

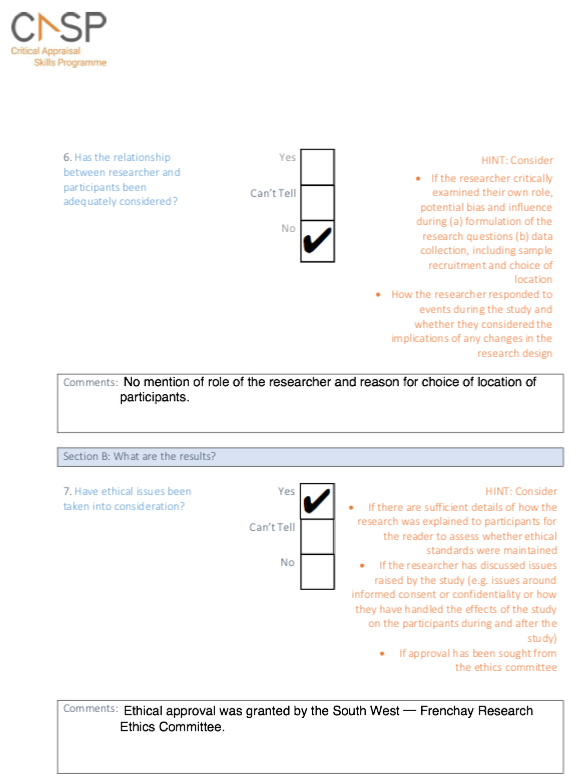

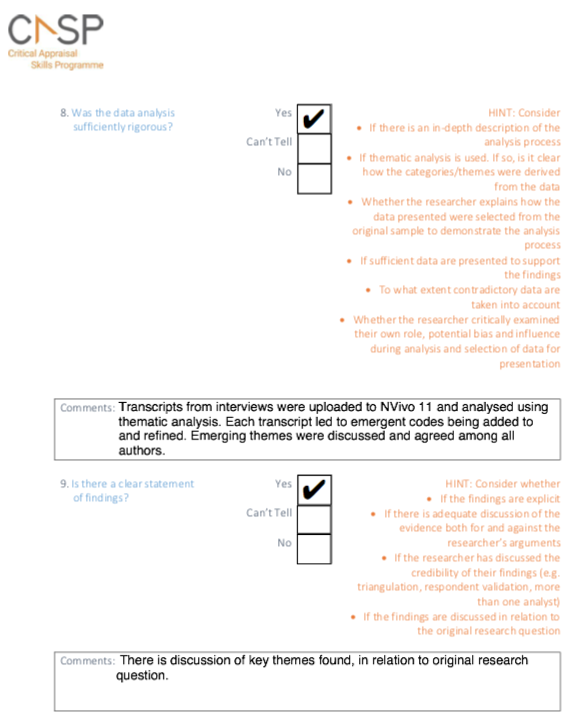

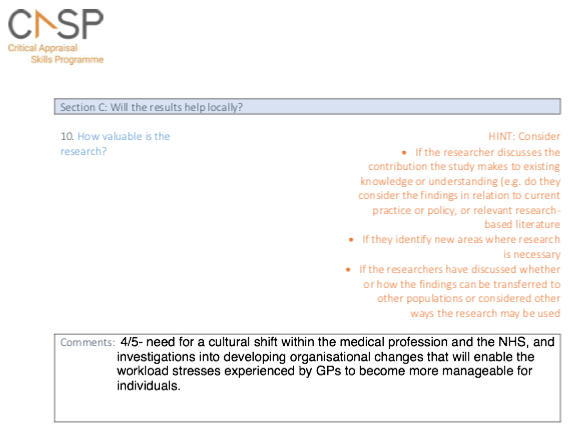


Teo et al 2021
Factors associated with self-reported burnout level in allied healthcare professionals in a tertiary hospital in Singapore


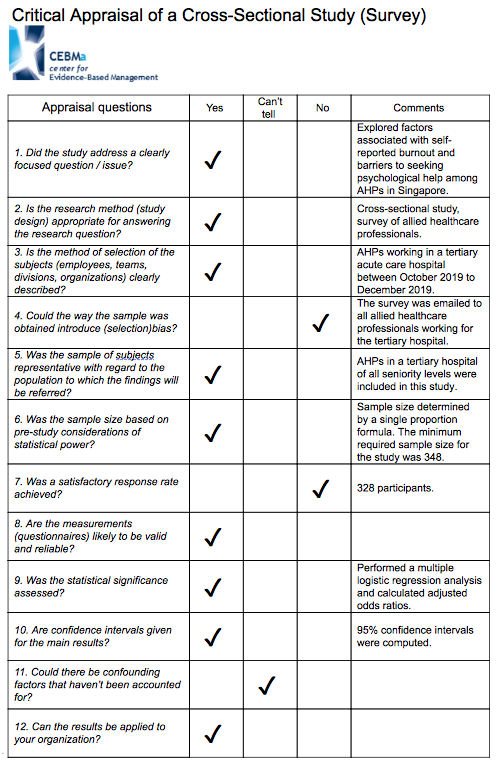


Whit et al 2018
Barriers to mental healthcare for psychiatrists


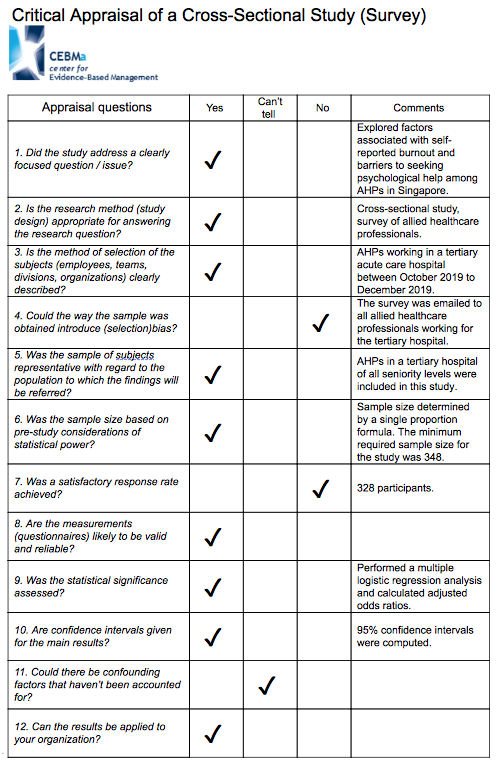


Worley et al 2008
Our fallen peers: A mandate for change


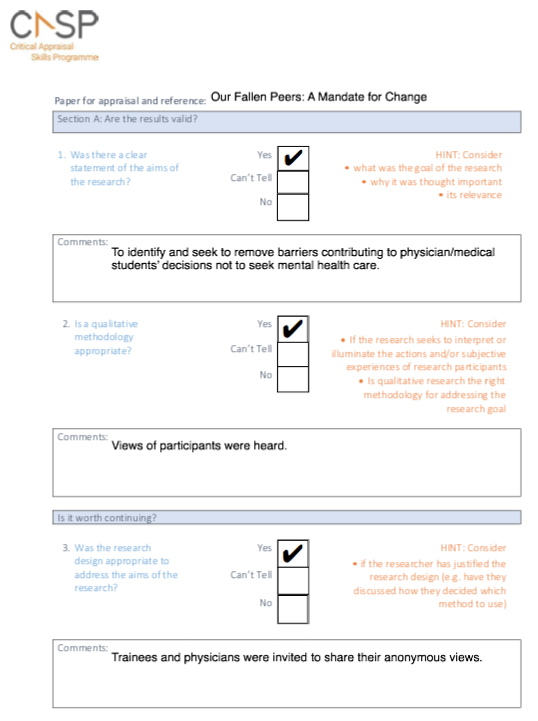


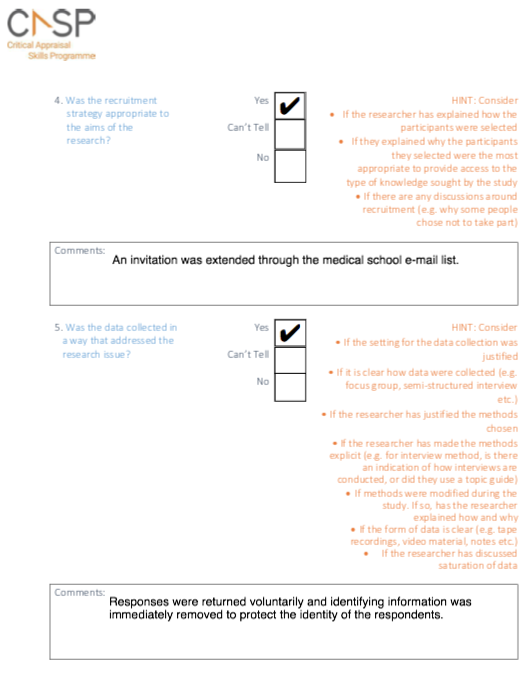

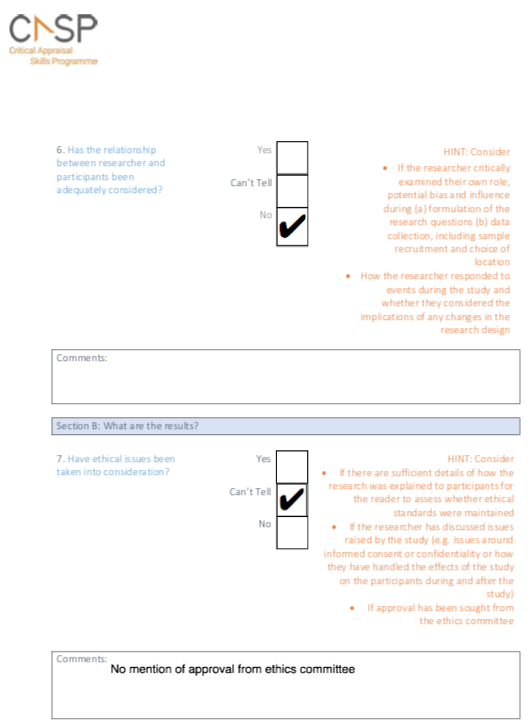

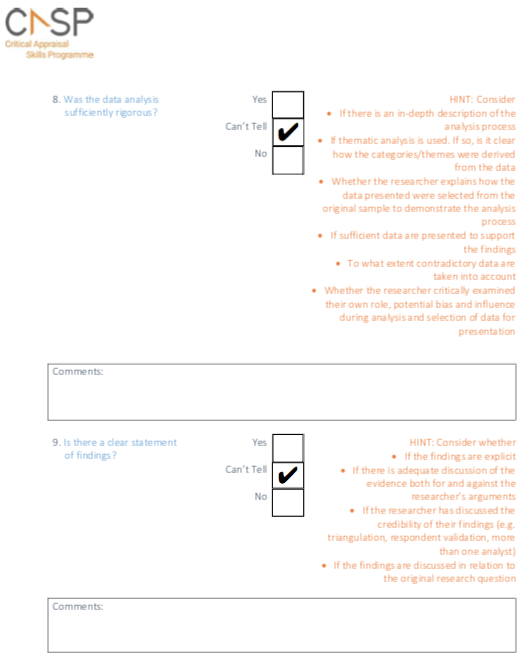

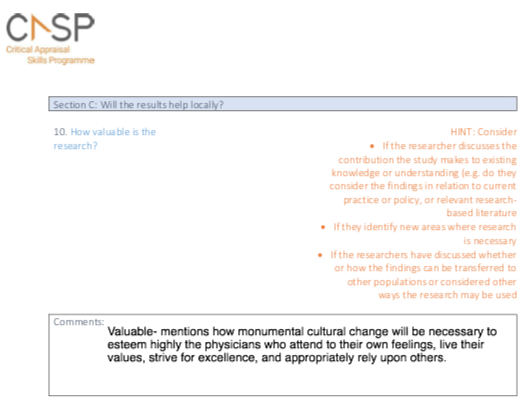

Supplement: Supplementary file 4 — Additional file 4. Critical appraisal checklists. [file 12888_2022_4202_MOESM4_ESM.docx]
